# Mapping the plasma metabolome to human health and disease in 274,241 adults

**DOI:** 10.1038/s42255-025-01371-1

**Published:** 2025-09-19

**Authors:** Jia You, Xi-Han Cui, Yi-Lin Chen, Yi-Xuan Wang, Hai-Yun Li, Yi-Xuan Qiang, Ji-Yun Cheng, Yue-Ting Deng, Yu Guo, Peng Ren, Yi Zhang, Yu He, Xiao-Yu He, Shi-Dong Chen, Ya-Ru Zhang, Yu-Yuan Huang, Ying Mao, Jian-Feng Feng, Wei Cheng, Jin-Tai Yu

**Affiliations:** 1 Department of Neurology and National Center for Neurological Disorders, Huashan Hospital, Institute of Science and Technology for Brain-Inspired Intelligence, State Key Laboratory of Medical Neurobiology and MOE Frontiers Center for Brain Science, Shanghai Academy of Natural Sciences (SANS), Shanghai Medical College, Fudan University, Shanghai, China; 2 Key Laboratory of Computational Neuroscience and Brain-Inspired Intelligence, Fudan University, Ministry of Education, Shanghai, China; 3 Department of Neurosurgery, Huashan Hospital Fudan University, Shanghai, China; 4 Department of Computer Science, University of Warwick, Coventry, UK

**Keywords:** Biomarkers, Diseases, Translational research, Metabolism

## Abstract

A systematic characterization of metabolic profiles in human health and disease enhances precision medicine. Here we present a comprehensive human metabolome–phenome atlas, using data from 274,241 UK Biobank participants with nuclear magnetic resonance metabolic measures. This atlas links 313 plasma metabolites to 1,386 diseases and 3,142 traits, with participants being prospectively followed for a median of 14.9 years. This atlas uncovered 52,836 metabolite–disease and 73,639 metabolite–trait associations, where the ratio of cholesterol to total lipids in large low-density lipoprotein percentage was found as the metabolite associated with the highest number (*n* = 526) of diseases. In addition, we found that more than half (57.5%) of metabolites showed statistical variations from healthy individuals over a decade before disease onset. Combined with demographics, the machine-learning-based metabolic risk score signified the top 30 (around 10%) metabolites as biomarkers, yielding favourable classification performance (area under the curve > 0.8) for 94 prevalent and 81 incident diseases. Finally, Mendelian randomization analyses provided support for causal relationships of 454 metabolite–disease pairs, among which 402 exhibited shared genetic determinants. Additional insights can be gleaned via an accessible interactive resource (https://metabolome-phenome-atlas.com/).

## Abstract (web summary)

Using data from the UK Biobank, the authors develop a comprehensive human metabolome–phenome atlas, including a browsable web tool, to uncover unique metabolite–trait and metabolite–disease associations with time, and discuss potential causal relationships.

## Main

Metabolites, signifying a complex interplay between genotype, behaviour and environment^1–3^, provide a unique readout of human health and disease^4,5^. Compared to other blood-based biomarkers, metabolites are more closely tied to phenotypes due to their key roles in physiological function control^6^. Therefore, accurately evaluating metabolic disturbance offers a comprehensive, precise and dynamic view of the disease status^6^. Furthermore, characterizing metabolic changes preceding disease diagnosis, especially before clinical manifestation, could help understand the aetiology and biological signatures^7,8^. Consequently, comprehending how metabolites interact with phenotype could assist in the discovery of robust biomarkers and therapeutic targets, facilitating personalized risk assessment and intervention, and advancing precision medicine.

Recent studies on metabolites have been pivotal in identifying biomarkers^9–11^, categorizing diseases^3,12,13^ and assessing risks^14,15^ for diseases. Most metabolomic studies have typically been cross-sectional with case–control approaches biased by possible reverse causality, thus failing to identify early metabolite signatures. Besides, they commonly focused on a limited number of disease outcomes. Consequently, many important questions remained to be solved to comprehensively interrogate the applicability of the metabolomes in a large, prospective, rich phenotypic cohort. For instance, what are the specific or shared metabolites among broad human phenotypes? What are the variational patterns of metabolites before disease onset, and when do these alterations occur? How will the metabolome facilitate disease discrimination across a comprehensive human disease spectrum? What are the potentially causal relationships between disease–metabolite pairs and any shared genetic determinants? Advancements in high-throughput metabolomics technologies^16,17^, when combined with large-scale phenotypic data, present an unparallelled opportunity to answer these questions by systematically mapping the connections between metabolites and human health and disease.

**Fig. 1:**
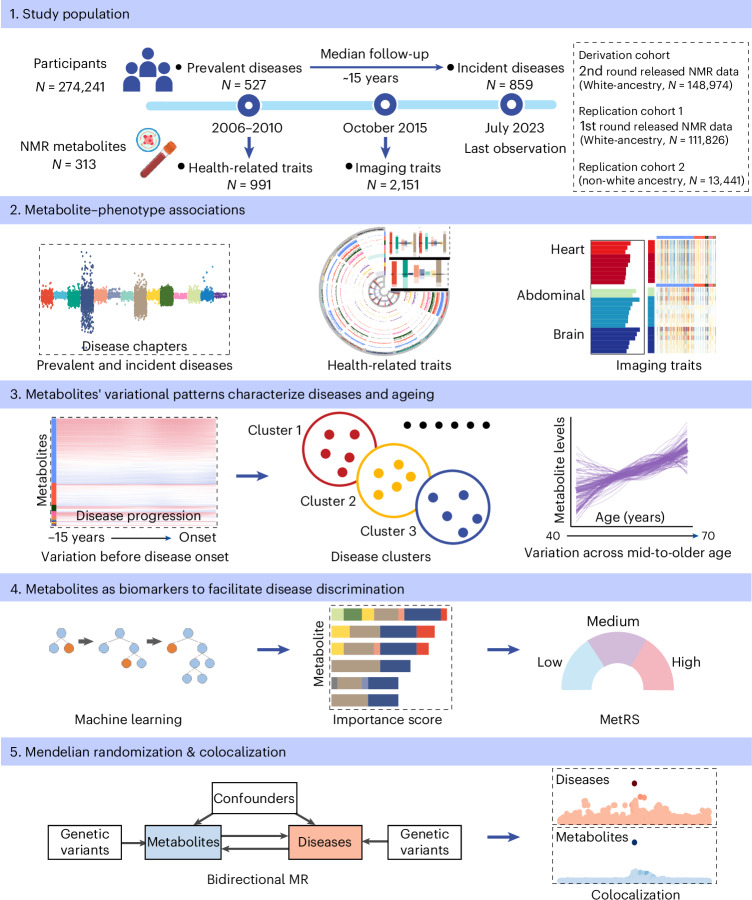
Study overview. First, we extracted data from 274,241 UKB participants with a median follow-up time of 14.9 years, including 313 NMR metabolites, 527 prevalent diseases and 859 incident diseases defined by three-character ICD-10 codes, 991 health-related traits and 2,151 imaging traits. The participants were then divided into three cohorts: derivation cohort, replication cohort 1 (white ancestry) and replication cohort 2 (non-white ancestry). Second, we established a human metabolome–phenome atlas by systematically examining over 1.4 million associations between metabolites and every phenotype. Third, the atlas depicted the metabolite variations assessed at different times before disease onset, and hierarchical clustering grouped diseases with similar variations before diagnosis. Next, machine-learning-based MetRS was used to quantify the capability of metabolic profiling for disease discrimination. Finally, bidirectional Mendelian randomization (MR) and colocalization analysis were conducted to reveal the causal relationship between metabolites and diseases and their sharing genetic determinants.

**Extended Data Fig. 1:**
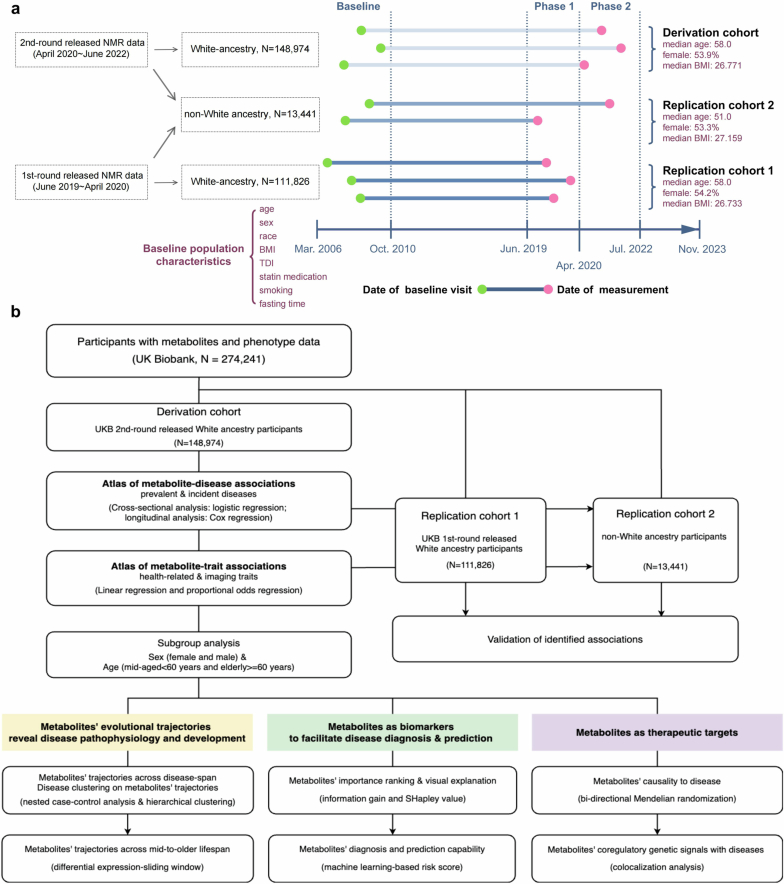
Study design. **(a)** Illustration of cohort characteristics and timeline design. **(b)** Analytical workflow.

This study established a human metabolome–phenome atlas by systematically examining over 1.4 million associations between 313 plasma metabolites and a comprehensive range of human phenotypes (comprising 1,386 diseases and 3,142 traits) across 274,241 prospectively followed (median 14.9 years) participants from the UK Biobank (UKB). In addition, the atlas depicted the metabolite variations assessed at different times before disease onset. Furthermore, machine-learning-based metabolic risk score (MetRS) aids in quantifying the capability of metabolic profiling for prevalent disease classification and incident disease prediction. Integrating genetic signals of metabolites and diseases, we inferred the potentially causal effects of metabolites upon diseases using Mendelian randomization analysis. This publicly available atlas (https://metabolome-phenome-atlas.com/) provides a comprehensive tool to better understand human health and disease through a metabolome readout. The detailed analytical workflow is depicted in Fig. 1 and Extended Data Fig. 1b.

## Results

### Population characteristics and phenotypes

**Extended Data Fig. 2:**
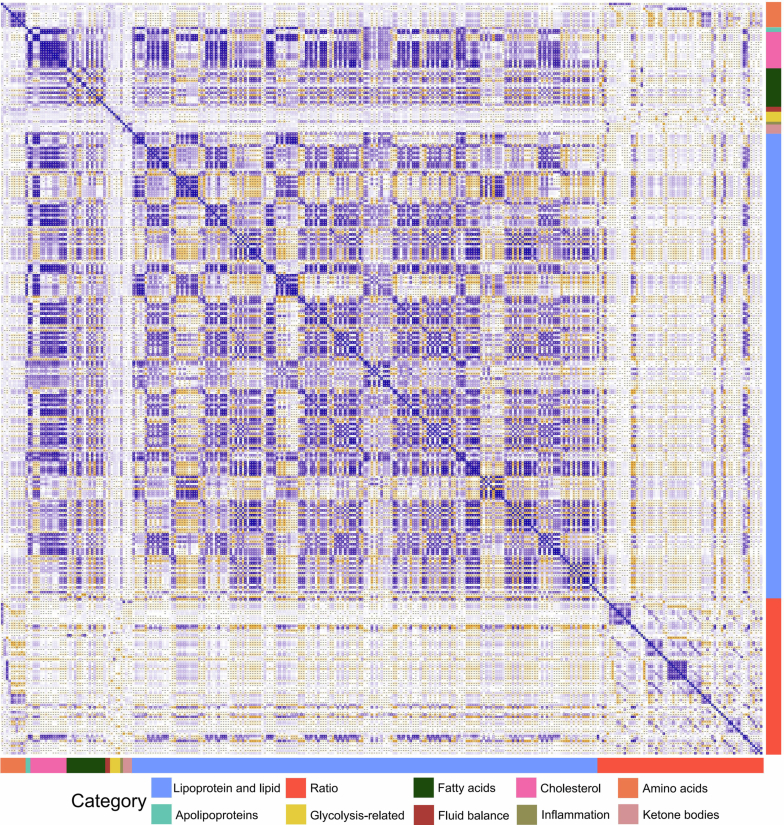
Spearman pairwise correlation coefficients across all metabolites. A heatmap showing Spearman correlation coefficients between all pairs of metabolites, organized by category. Each pixel represents the correlation between two metabolites, with the color scale indicating the strength and direction of the correlation. Metabolites are grouped and color-coded by category along the bottom and side bars. Source data

This study included 274,241 participants from the UKB with nuclear magnetic resonance (NMR) metabolic measures. Analysed participants had a median age of 58.0 (interquartile range, 50.0–63.0) years at the time of blood sample collection, of whom 54.0% (*n* = 147,994) were females, and 95.1% (*n* = 260,800) were of white ancestry (Supplementary Table 1). Until November 2023, participants had a median follow-up of 14.9 (interquartile range, 14.1–15.5) years. Blood samples were collected at participants’ baseline visits, and 313 NMR metabolic profiles were measured (Supplementary Table 2). The metabolites’ correlation is shown in Extended Data Fig. 2. Our analysis revealed strong positive correlations, particularly within lipid-related metabolites, reflecting shared biological pathways, while weaker correlations were observed between lipid metabolites and non-lipid classes, such as amino acids and organic acids. The participants were partitioned into three cohorts: a derivation cohort of 148,974 individuals of white ancestry in phase 2, a replication cohort 1 of 111,826 individuals of white ancestry from phase 1 and a replication cohort 2 of 13,441 individuals of non-white ancestry from both phases (Extended Data Fig. 1a).

**Fig. 2:**
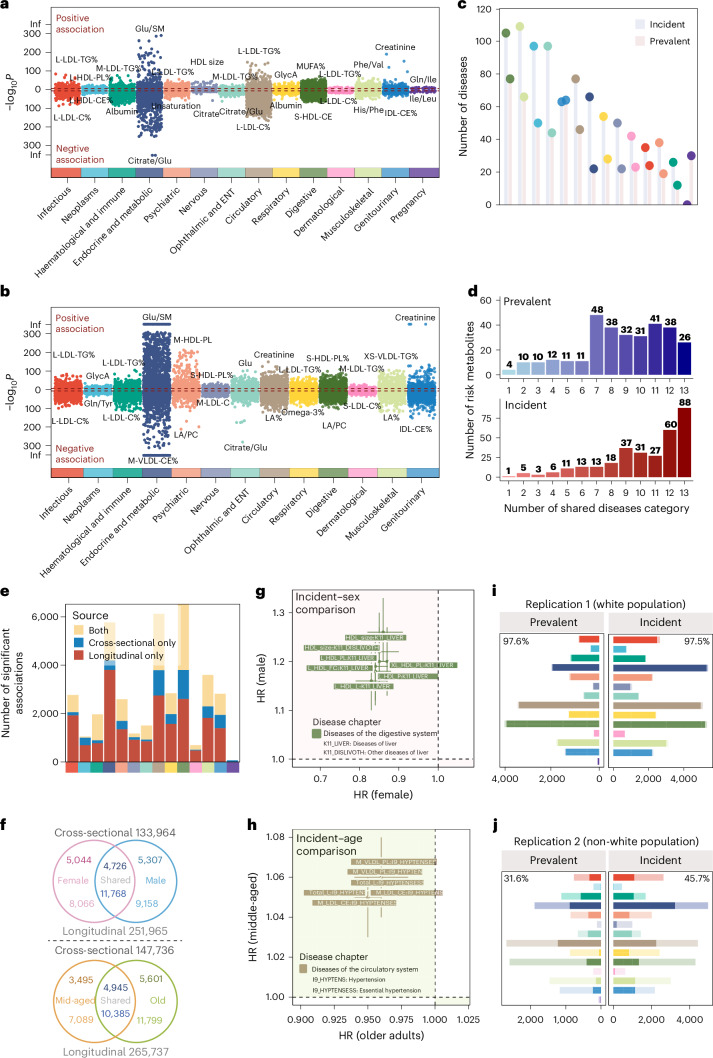
Atlas of metabolite–disease association analysis results. **a**,**b**, Metabolite–disease associations were revealed by logistic regression (**a**) and the Cox proportional-hazards model (**b**), coloured by the ICD-10-based disease chapter. Positive associations (OR > 1 or HR > 1) are above the red dashed line; negative associations (OR < 1 or HR < 1) are below. Only statistically significant associations passing Bonferroni-corrected thresholds are shown: (**a**) *n* = 18,594, *P* < 3.03 × 10^⁻7^ (0.05/(313 × 527)); (**b**) *n* = 34,242, *P* < 1.86 × 10⁻^7^ (0.05 / (313 × 859)). M-LDL-TG%, triglycerides to total lipids in medium low-density lipoprotein percentage; L-HDLPL%, phospholipids to total lipids in large high-density lipoprotein percentage; IDL-CE%, cholesteryl esters to total lipids in intermediate-density lipoprotein percentage; S-HDL-CE, cholesteryl esters in small high-density lipoprotein; MUFA%, monounsaturated fatty acids to total fatty acids percentage; M-VLDL-CE%, cholesteryl esters to total lipids in medium very low-density lipoprotein percentage; M-HDL-PL, phospholipids in medium highdensity lipoprotein; M-LDL-C, cholesterol in medium low-density lipoprotein; S-LDL-C%, cholesterol to total lipids in small low-density lipoprotein percentage; LA%, linoleic acid to total fatty acids percentage; LA/PC: Linoleic acid to phosphatidylcholines ratio; XS-VLDL-TG%, triglycerides to total lipids in extra-small very low-density lipoprotein percentage. ENT, ear, nose and throat. **c**, Number of prevalent and incident diseases by disease chapter. **d**, Distribution of risk metabolites across disease categories. The *x* axis represents the number of shared disease categories (1 to 13). The *y* axis shows the number of associated metabolites. **e**, Comparison of the number of associations in cross-sectional, prospective analysis, or both, with a colour bar indicating different disease categories. **f**, Venn diagram of subgroup analyses by sex (top) and age (bottom). Numbers outside circles show total associations tested; upper numbers are for cross-sectional analysis, while lower numbers are for prospective analysis. **g**,**h**, Metabolite–disease pairs with inconsistent directions of effect in female versus male (**g**) and middle-aged versus older (**h**) individuals in a prospective subgroup analysis, coloured by disease chapter. Error bars represent 95% CIs. **i**,**j**, Validation of metabolite–disease associations from the derivation cohort in replication cohort 1 (**i**) and replication cohort 2 (**j**). Bars, coloured by disease chapter, show statistically meaningful associations in the derivation cohort, with the darker portion indicating those with *P* < 0.05 in replication cohorts. The top corner numbers represent the replication proportions within each facet. Source data

**Extended Data Fig. 3:**
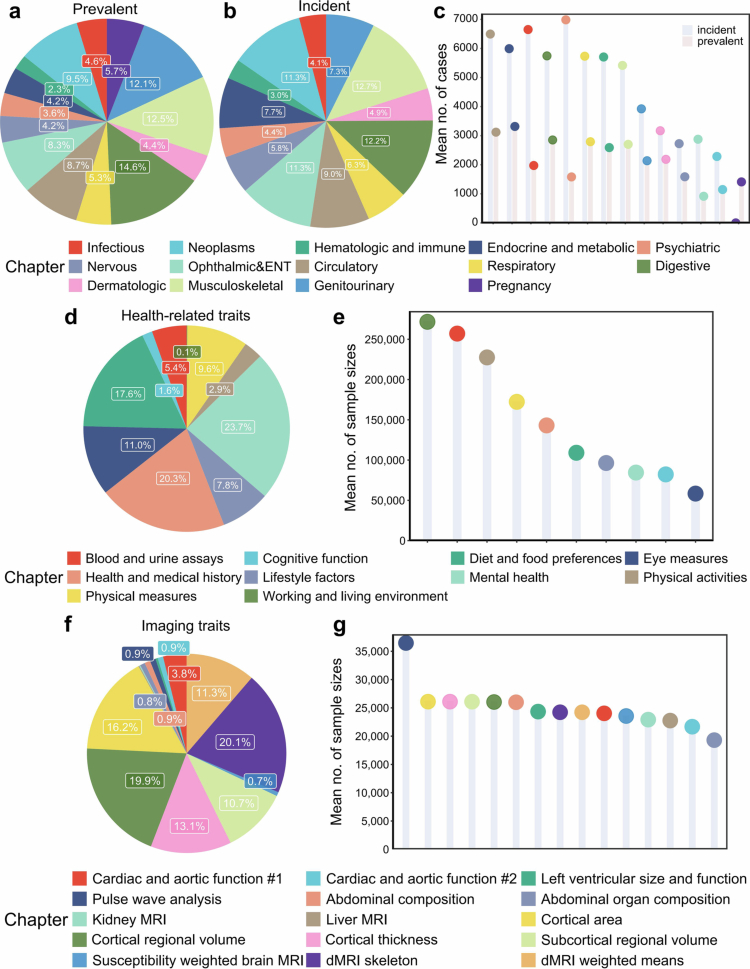
Distribution and sample sizes of diseases and traits. (**a-b**) Pie chart illustrating the distribution of diseases, showing **(a)** prevalent diseases and **(b)** incident diseases, each is classified according to disease chapters. **(c)** The average number of cases for prevalent and incident diseases is classified by disease chapter. **(d)** Pie chart displaying the distribution of health-related traits, which are categorized according to chapters derived from UK Biobank paths. **(e)** The average number of sample sizes for health-related traits was classified by trait chapter. **(f)** Pie chart displaying the distribution of imaging traits, classified by imaging trait chapter. **(g)** The average number of sample sizes of imaging traits classified by imaging trait chapter. Source data

Phenotypes adopted in the study comprise two main categories of diseases and traits. The diseases, defined using International Classification of Diseases (ICD)-10 codes, were classified into prevalent diseases (*n* = 527, diagnosed before the blood collection) and incident diseases (*n* = 859, diagnosed after the blood collection; Supplementary Tables 3 and 4). Specifically, for prevalent diseases, digestive diseases (*n* = 77, 14.6%), musculoskeletal diseases (*n* = 66, 12.5%) and genitourinary diseases (*n* = 64, 12.1%) emerge as the three most prominent disease categories (Fig. 2c and Extended Data Fig. 3a). The average number of cases per disease chapter varied between 2,725 and 6,984 (Extended Data Fig. 3c). As for incident diseases, musculoskeletal diseases (*n* = 109, 12.7%), digestive diseases (*n* = 105, 12.2%) and neoplasms (*n* = 97, 11.3%) are the top three categories (Fig. 2c and Extended Data Fig. 3b,c).

Traits were composed of health-related traits (*n* = 991) and imaging traits (*n* = 2,151; Supplementary Tables 5 and 6). Health-related traits were classified into ten chapters, with mental health (*n* = 235) and health and medical history (*n* = 201) being the top two categories regarding the amount (Extended Data Fig. 3d,e). Imaging traits were extracted from brain magnetic resonance imaging (MRI; *n* = 1,978), heart MRI (*n* = 129) and abdominal MRI (*n* = 44; Extended Data Fig. 3f,g).

### Atlas of metabolite–disease associations

Relationships between metabolites and diseases were analysed cross-sectionally (logistic regressions) and prospectively (Cox proportional-hazards regressions) for prevalent and incident diseases, respectively. For cross-sectional analysis, 18,594 (11.3%) metabolite–disease associations were identified at the Bonferroni-corrected threshold of 3.03 × 10^−7^ (0.05/(313 × 527)), where haematologic and immune diseases (31.8% = 1,196/(313 × 12)) constituted the highest proportion, followed by endocrine and metabolic diseases (28.6%; Fig. 2a and Supplementary Table 7). The prospective analysis identified 34,242 (13.2%) associations under *P* < 1.86 × 10^−7^ (0.05/(313 × 859)), where endocrine and metabolic diseases made up the largest proportion of 26.0% (5,378/(313 × 66)), followed by infectious diseases (24.1%; Fig. 2b and Supplementary Table 8). Less than half (42.5%) of associations were concurrently observed as statistically significant in cross-sectional and prospective analyses (Fig. 2e).

In cross-sectional analysis, 43.5% (*n* = 136) of metabolites were associated with ≥10 disease chapters, while in prospective analysis, nearly two-thirds (65.8%, *n* = 206) were associated with ≥10 disease chapters (Fig. 2d). Notably, the ratio of cholesterol to total lipids in large low-density lipoprotein (LDL) particles (L-LDL-C%) and the ratio of triglycerides to total lipids in large LDL% (L-LDL-TG%) were the top two disease-associated metabolites, and they were found associated with 203 and 188 prevalent diseases and 323 and 317 incident diseases, respectively (Supplementary Table 11).

In subgroup analyses of sex and age (<60 and ≥60 years), metabolome-disease heterogeneity was observed, with nearly half of the associations identified uniquely. Specifically, for sex, 4,726 cross-sectional associations were identified in both females and males, while 10,351 were uniquely found in females (*n* = 5,044) and males (*n* = 5,307). For age, 4,945 cross-sectional associations were shared, with 9,096 being unique (Fig. 2f). Notably, seven associations with divergent effect directions were explicitly identified in diseases of the digestive system, differing between females and males. For instance, high-density lipoprotein (HDL) size, total lipids in large HDL (L-HDL-L) and free cholesterol in large HDL (L-HDL-FC) exhibited protective effects against liver diseases in females (hazard ratio (HR; 95% confidence interval (CI)) = 0.86 (0.82–0.91), 0.83 (0.79–0.87) and 0.84 (0.80–0.88), respectively), but were associated with increased risk in males (HR (95% CI) = 1.26 (1.20–1.33), 1.16 (1.10–1.22) and 1.19 (1.12–1.25), respectively; Fig. 2g and Supplementary Table 12). The age-stratified subgroup analysis revealed six associations in the prospective analysis had inconsistent effects, all within the circulatory system. For instance, total lipids in lipoprotein particles (Total-L), cholesteryl esters in medium LDL (M-HDL-CE) and phospholipids in medium VLDL (M-VLDL-PL) were risk factors for middle-aged but protective for older individuals (Fig. 2h and Supplementary Table 13). Interaction analyses between metabolites and sex, as well as metabolites and age, revealed consistent findings with subgroup analysis, that only a small number of associations exhibited interactions with age or sex across phenotype groups (Supplementary Tables 7 and 8).

### Atlas of metabolite–trait associations

Next, relationships between metabolites and traits were examined, which included 991 health-related traits and 2,151 imaging traits. The health-related traits were derived from physical measurements, questionnaires, and blood and urine assays of UKB participants at recruitment. The imaging traits were extracted from MRI scanning at the imaging visit. Although these traits are not disease diagnosis information, they may be closely related to body health by acting as risk factors, clinical manifestations signifying disease status, and others.

**Fig. 3:**
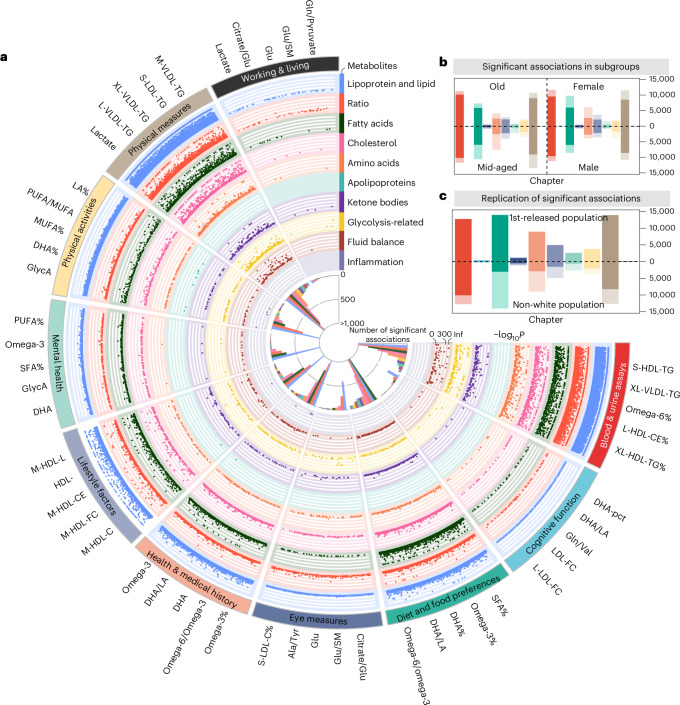
Atlas of metabolite–trait association analysis results. **a**, Fuji plot of associations between metabolites and health-related traits (*n* = 62,887). This circular plot displays statistically meaningful associations identified between metabolites and health-related traits. The outer ring lists the top five metabolites with the most statistically significant associations per trait category. Each dot represents a statistically meaningful association, with categories labelled around the perimeter. Colours indicate different metabolite categories. **b**, Associations between metabolites and health-related traits in subgroup analyses. Bars are coloured by trait categories, with darker segments indicating associations common to both sex or age subgroups. Numbers show the proportion of shared associations within each subgroup. **c**, Validation of associations in the replication cohort 1 (upper) and 2 (lower). Bars, coloured by trait categories, represent statistically meaningful associations in the derivation cohort. Darker segments indicate associations with *P* < 0.05 in replication cohorts. Numbers show the replication rate within each cohort. PUFA, polyunsaturated fatty acid; MUFA, monounsaturated fatty acid; DHA, docosahexaenoic acid. Source data

We found 62,887 associations (20.3% = 62,887/(991 × 313)) for health-related traits under a Bonferroni-corrected *P* < 5.08 × 10^−8^ (0.05/(313 × 3,142)). Associations were found mainly in diet and food preferences (*n* = 14,050, 22.3%) and physical measurements (*n* = 13,986, 22.2%). The high light scatter reticulocyte counts in blood and urine assays were found to be associated with the most metabolites (*n* = 278, 88.9%), where the omega-6 fatty acids to total fatty acid percentage (omega-6%) (regression coefficient *β* = −19.34, *P* < 1 × 10^−300^) and triglycerides in small HDL (*β* = 19.77, *P* < 1 × 10^−300^) showed the most pronounced associations. Among metabolite categories, metabolic ratios (*n* = 9,903) and lipoproteins and lipids (*n* = 8,660) were found to have the largest number of associations (Fig. 3a and Supplementary Table 9).

**Extended Data Fig. 4:**
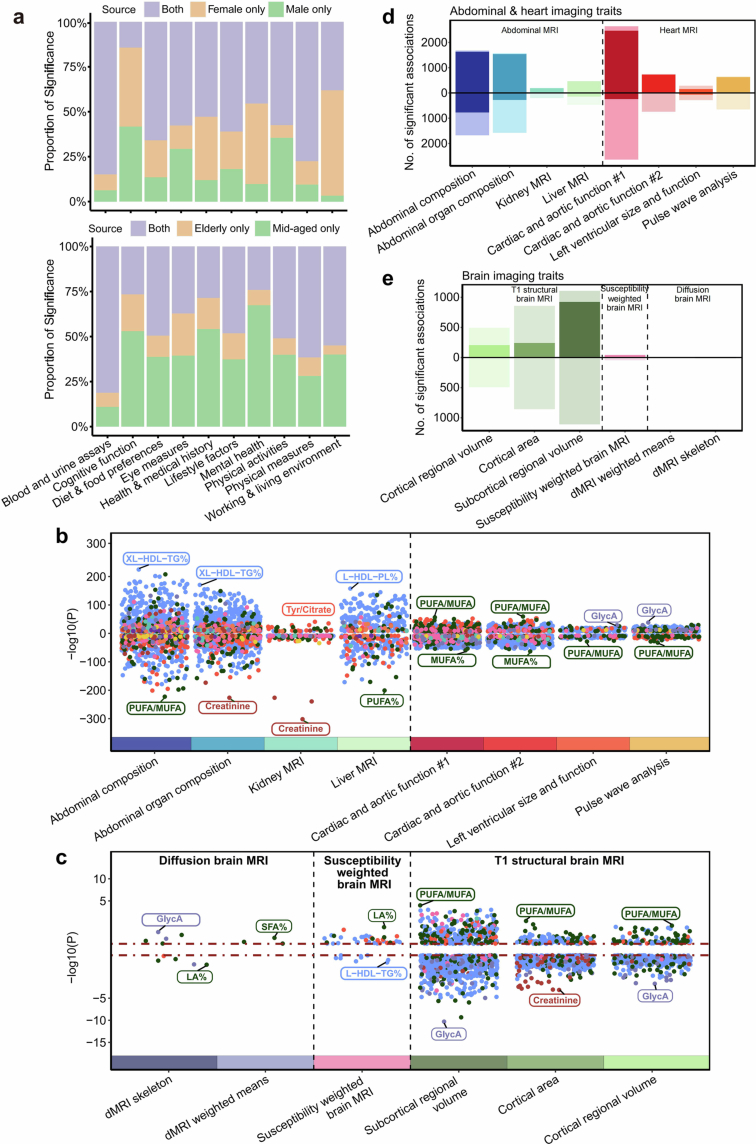
Summary of metabolite-trait association analysis results. **(a)** Proportion of shared and specific associations observed in subgroup analyses based on sex (upper panel) and age (lower panel) for health-related traits. (**b, c**) Statistically significant associations between metabolites and imaging traits related to the abdomen and heart **(b)** and brain **(c)**, colored by metabolite category. The upper section indicates positive associations (with OR > 1 or HR > 1), while the lower section denotes negative associations (with OR < 1 or HR < 1). Only statistically significant associations passing Bonferroni-corrected thresholds are shown: P < 5.08×10-8 (0.05/[313*3,142]). (**d, e**) Replication of associations between metabolites and abdominal and heart imaging traits **(d)** and brain imaging traits **(e)** identified in the derivation cohort, replicated in the replication cohort (upper panel) and the non-White cohort (lower panel). Bars were colored by trait categories, representing the number of associations in the derivation cohort. The darker portion of the bar indicates the number of these associations that had Bonferroni corrected P < 0.05 in replication cohorts. HR, hazard ratio; OR, odds ratio. Source data

Metabolite–trait associations identified in the discovery cohort were successfully replicated in the subgroup analysis across various demographics: 66.9% in females, 77.3% in males, 81.7% in older adults and 61.1% in middle-aged individuals. Among these, 542 associations demonstrated different effect directions in sex (*n* = 198) and age (*n* = 344) subgroups (Fig. 3b, Extended Data Fig. 4a and Supplementary Tables 13 and 14). For instance, 31 lipoproteins and lipids associated with alcohol consumption exhibited opposite directional associations between females (negative) and males (positive), aligning with previous studies indicating that influential factors such as body composition, hormonal levels and genetic predispositions play critical roles in how men and women metabolize and respond to alcohol differently^18^. Furthermore, 47 metabolomes associated with cognitive function exhibited opposite effects between middle-aged (positive) and older (negative) groups, as supported by previous research highlighting the complexity of metabolic influences on cognitive function across the lifespan, including variations in brain metabolism, vascular health and oxidative stress accumulation^19^.

**Fig. 4:**
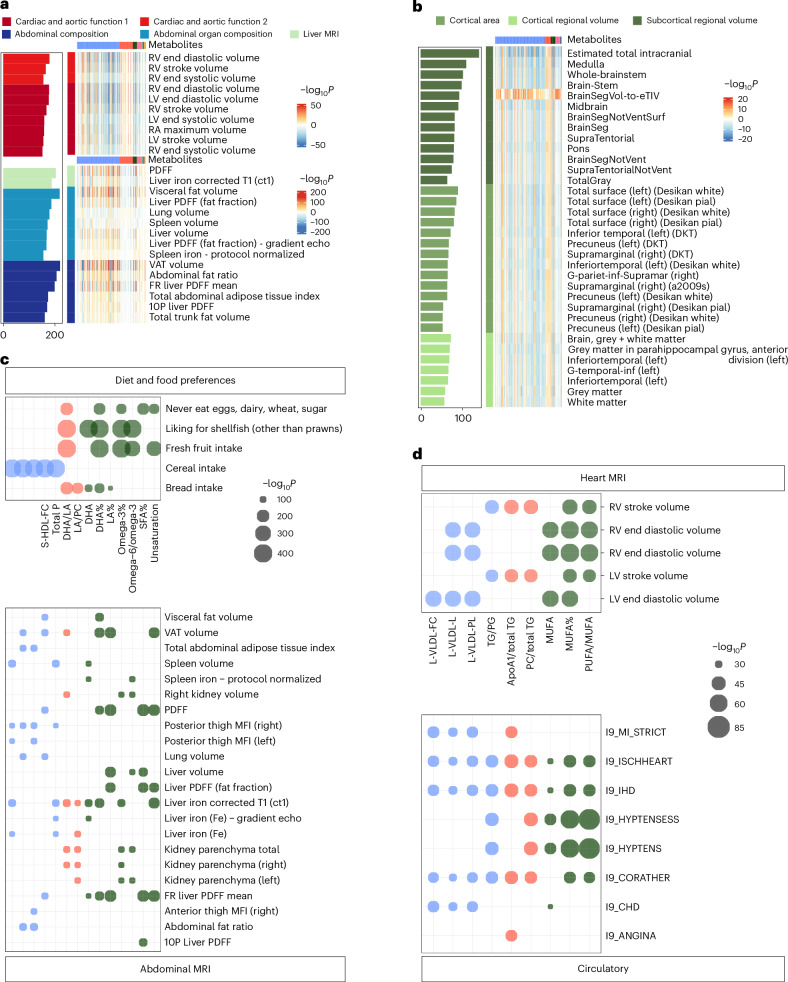
Integrated atlas of metabolite–imaging trait associations and shared associations with diseases. **a**,**b**, Associations between metabolites and imaging traits. Associations with abdomen and heart imaging (**a**) and brain imaging (**b**) traits. Rows represent specific imaging traits, with a colour gradient indicating significance levels (darker colours denote higher significance). The bar plot on the left shows the count of statistically meaningful metabolites per imaging trait. Statistical significance was defined using a Bonferroni-corrected threshold of *P* < 5.08 × 10^−8^ (0.05/(313 × 3,142)). **c**,**d**, Shared metabolites between imaging and health-related traits or diseases. **c**, Shared metabolites between abdominal imaging traits and diet and food preferences. **d**, Shared metabolites between heart imaging traits and various incident circulatory diseases. Point size indicates the magnitude of *P* values, with larger points representing more statistically meaningful associations (smaller *P* values). Bonferroni-adjusted significance thresholds were *P* < 5.08 × 10^−8^ (0.05/(313 × 3,142)) for imaging and health-related traits, and *P* < 1.86 × 10^⁻7^ (0.05/(313 × 859)) for incident diseases. LV, left ventricle; MFI, muscle fat infiltration; PDFF, proton density fat fraction; DKT, Desikan-Killiany-Tourville; FR, fat referenced; RV, right ventricle; VAT, visceral adipose tissue; RA, right atrium; PG, phosphoglycerides; TG, triglycerides. Source data

For imaging traits, 10,752 statistically significant associations were identified under *P* < 5.08 × 10^−8^ (Fig. 4a,b and Supplementary Table 10). The associations were primarily found in cardiac and aortic function (*n* = 2,636). The most notable associations were creatinine to kidney MRI (*β* = −8.626, *P* = 4.58 × 10^−221^) and triglycerides in very large HDL (XL-HDL-TG%) to abdominal organ composition (*β* = −0.132, *P* = 2.17 × 10^−219^), respectively (Extended Data Fig. 4b). In brain imaging, metabolites exhibited relatively stronger associations with T1 structural brain MRI, and the most statistically meaningful associations included glycoprotein acetyls (GlycA; *β* = −7.868, *P* = 4.89 × 10^−17^) and polyunsaturated fatty acids/monounsaturated fatty acids (*β* = −7.894, *P* = 4.58 × 10^−16^) to subcortical regional volume (Extended Data Fig. 4c).

### The metabolome bridges the relationship between diseases and traits

Fourteen metabolites exhibited associations with both diet and food preferences and abdominal MRI analysis. Additionally, heart MRI analysis revealed nine common metabolites associated with circulatory system diseases (Fig. 4c,d and Supplementary Tables 15 and 16). Besides, we calculated the distribution of metabolites according to their categories across different disease and trait groups (Supplementary Tables 17–20). The results revealed that lipoproteins and lipids were the most frequently associated metabolite categories with both diseases and traits. Fatty acids and metabolite ratios emerged as the second and third most frequently associated categories, respectively.

In addition, we included estimated glomerular filtration rate (eGFR) as an additional covariate in the regression models. The results indicated that most of the associations across different phenotype categories (90.0% in incident diseases, 87.1% in prevalent diseases, 92.7% in the health-related traits and 76.0% in imaging traits) remained statistically meaningful (Supplementary Tables 7–10), indicating the associations between metabolites and disease/traits were not largely impacted by eGFR.

### Replication of metabolite–phenotype associations

To validate the associations identified, we conducted replication analysis separately in white and non-white ancestry groups, using a statistically meaningful threshold determined by the Benjamini–Hochberg procedure. The white ancestry population verified the majority findings of 18,148 (97.6%) and 33,396 (97.5%) associations were successfully replicated for cross-sectional and prospective metabolite–disease analysis, respectively. In contrast, for the non-white population, 4,638 (31.6%, among 14,684 associations available to validate) and 14,096 (45.3%, among 31,103 associations available to validate) associations were replicated, indicating a notable heterogeneity between the two ancestry groups (Fig. 2i,j).

The replication of health-related and imaging traits in the white population examined 73,639 available associations, and 71,613 (97.2%) were successfully verified. The non-white population exhibited a much lower proportion of 42.0%, where 30,975 associations were validated among 73,639 available ones (Fig. 3c and Extended Data Fig. 4d,e).

### Metabolite variation assessments at different times characterize disease progression

**Extended Data Fig. 5:**
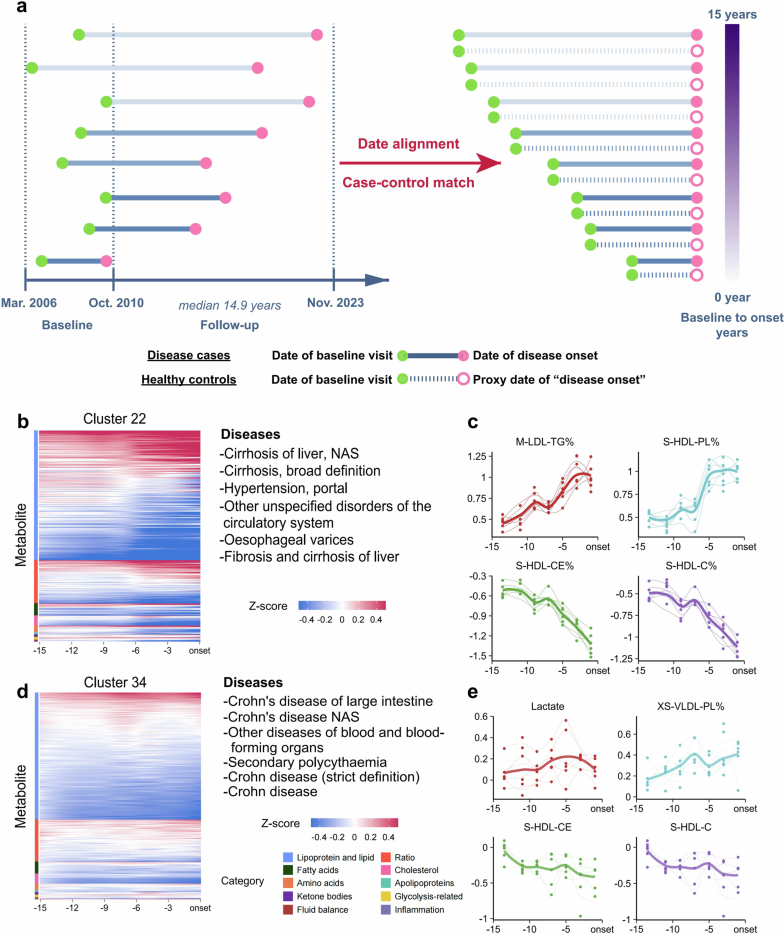
Metabolites assessed at different time before disease onset. **(a)** Illustrative graph of the nested case-control study design, where each disease case was matched with five controls. The index date for each case is aligned, and for healthy controls, it is assumed that they have the same ‘proxy onset dates’ as their matched cases. Various timeframes preceding disease diagnosis, ranging from 15 years before onset to the disease onset itself, are considered. Within these timeframes, the relative levels of metabolites in cases are compared to those in controls, allowing to depict metabolites’ variation before disease onset. (**b, d**) The heatmaps depict representative Cluster 22 **(b)** and Cluster 34 **(d)** of incident diseases, grouped based on hierarchical clustering using metabolite levels assessed at different timeframes before diseases. Metabolite levels have been Z-scored and estimated using the LOESS method to standardize the data. (**c, e**) The line plots show the Z-scored levels of selected metabolites over the 15 years preceding disease onset in Cluster 22 **(c)** and Cluster 34 **(e)**. The selected metabolites include the two most positively and two most negatively associated with the diseases. Each line represents the variational patterns of a specific disease, with thicker lines indicating the averaged patterns for all diseases within a particular cluster. In both panels, the x-axis represents the years before disease onset, and the y-axis represents the relative metabolite levels. The Z-score transformation was performed by subtracting the mean value of the controls and dividing by the variance of the controls to standardize the data. Source data

To uncover the variations in metabolite levels at different times before disease diagnosis, we delineated their variational patterns 15 years preceding the disease onset. We leveraged a nested case–control design in which the disease onset date for each case was aligned, and healthy controls were assumed to have the same proxy onset dates as their matched cases (Extended Data Fig. 5a). Among 34,242 statistically meaningful metabolite–disease pairs discovered in prospective association analysis, metabolites in 19,691 (57.5%) pairs emerged with variations a decade preceding disease onset, where L-LDL-TG% was found in the largest number of diseases (*n* = 246). In the meantime, metabolites in 10,275 (30.0%) pairs started to vary within 5 years before disease onset, and the degree of unsaturation in fatty acids accounted for the most diseases (*n* = 96; Supplementary Table 21).

**Fig. 5:**
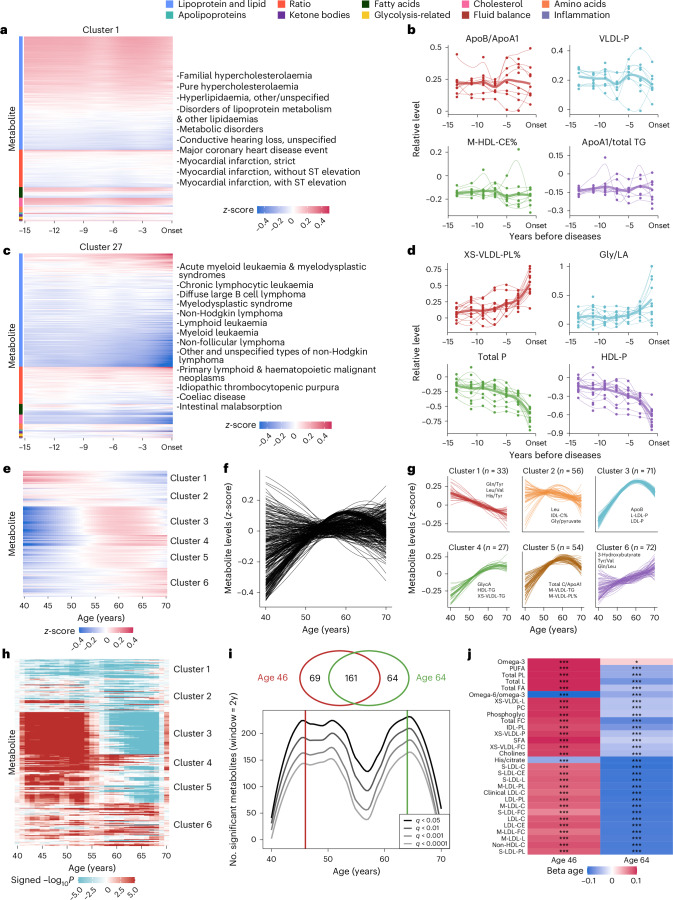
Assessment of metabolite variations at different times characterizes diseases and ageing. **a**,**c**, The heat maps depict representative cluster 1 (**a**) and cluster 27 (**c**) of incident diseases, grouped based on hierarchical clustering using metabolite levels assessed at different timeframes before diseases. Metabolite levels have been *z*-scored and estimated using the LOESS method to standardize the data. **b**,**d**, The line plots show the *z*-scored levels of selected metabolites over the 15 years preceding disease onset in cluster 1 (**b**) and cluster 27 (**d**). The selected metabolites include the two most positively and two most negatively associated with the diseases. Each line represents the variational patterns of a specific disease, with thicker lines indicating the averaged patterns for all diseases within a particular cluster. The *x* axis represents the years before disease onset, and the *y* axis represents the relative metabolite levels. The *z*-score transformation was performed by subtracting the mean value of the controls and dividing by the variance of the controls to standardize the data. **e**, Heat map of plasma metabolite levels across ages. The *x* axis represents age in years; the *y* axis lists individual metabolites. Blue indicates lower levels, and red indicates higher levels. **f**, The metabolite variations during ageing. Each line represents the *z*-scored levels of an individual metabolite across age. Lines were fitted using the LOESS method. **g**, The metabolite variations for six clusters based on similar patterns defined by hierarchical clustering. The *x* axis shows the age (40–70 years), and the *y* axis shows *z*-scored metabolite levels. Thin lines represent individual trajectories, and thick lines indicate cluster averages. The number of metabolites in each cluster is indicated, and the most statistically meaningful metabolites are plotted. **h**, Heat map illustrating the waves of ageing metabolites. The colour scale represents signed −log_10_*P*. **i**, The line plot shows the number of metabolites (*y* axis) as a function of age (*x* axis), with peaks at ages 46 and 64 derived from the DE-SWAN method. Lines represent different significance levels (*q* < 0.05, *q* < 0.01, *q* < 0.001, *q* < 0.0001). Venn diagram shows intersections of metabolites at peak ages. **j**, Top 15 metabolites identified at ages 46 and 64. Red and blue represent local increases and decreases, respectively. *q < 0.01, ***q < 0.001. LOESS, locally estimated scatterplot smoothing. Source data

To illuminate the metabolite variational patterns, diseases were grouped into 44 clusters based on the *z*-score-transformed metabolic measurements across multiple time intervals before diagnosis (Supplementary Table 22). There were seven clusters with more than 30 diseases and eight clusters with only one disease. Specifically, cluster 1 encompassed metabolic and cardiovascular disorders and auditory impairment (Fig. 5a). These disorders shared underlying biological mechanisms like lipid dysregulation and atherosclerotic processes, often exacerbated by systemic inflammatory responses^20^ and endothelial dysfunction^21^. Cluster 27 comprised a spectrum of haematological malignancies, associated disorders and other primary lymphoid and haematopoietic neoplasms (Fig. 5c). These conditions were characterized by haematopoiesis and immune regulation disruptions, often linked to genetic mutations and immune dysfunctions^22^.

We highlighted the changes of representative metabolites within each cluster to elucidate their shared patterns preceding disease onset. For cluster 1, the ratio of metabolites apolipoprotein B to apolipoprotein A1 (ApoB/ApoA1) and cholesteryl esters in medium HDL (M-HDL-CE) exhibited constant differences above or below the reference level of 0 throughout the 15 years (Fig. 5b). For cluster 27, phospholipids to total lipids in very small VLDL percentage (XS-VLDL-PL%) and glycine/linoleic acid (Gly/LA) remained stable until 5–10 years, while they were observed to have noticeable upward trends 5 years before onset. Meanwhile, total concentration of lipoprotein particles (Total P) and concentration of HDL particles (HDL-P) witnessed a decline during the 15-year period before disease onset (Fig. 5d). In addition, clusters 22 and 34 were demonstrated as examples of clusters sharing similar metabolite variation before disease onset, offering critical insights into the temporal dynamics of disease progression (Extended Data Fig. 5b,e).

### Metabolites assessed at different ages reflect ageing patterns

**Extended Data Fig. 6:**
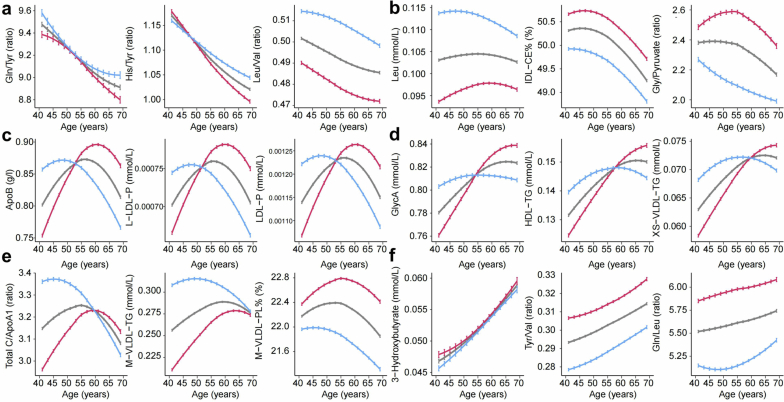
Representative metabolites’ variational patterns across mid-to-older ages. (**a–f**) Representative variational patterns of selected metabolites corresponding to six clusters from Fig. 5g. These metabolites were chosen based on their statistical significance, specifically those with the smallest P values during age regression analysis within each cluster. The figure utilizes color coding to represent different populations: grey for the overall population, red for females, and blue for males. The x-axis spans from 40 to 70 years, representing age, while the y-axis indicates the relative level of each metabolite. The lines depict the average metabolite levels, and the error bars represent standard errors, providing a measure of variability in metabolite levels across the age range. Source data

Considering age as the foremost indicator of human health, we investigated the variations of metabolites assessed at different ages during individuals’ baseline visits. *z*-scored metabolic measurements were grouped into six clusters with distinct variational patterns across 40 to 70 years (Fig. 5e–g and Supplementary Table 23). Specifically, metabolites that fell in clusters 1 and 6 were observed to have monotonic trends of consistent increase and decrease, respectively. Clusters 3, 4 and 5 exhibited obvious nonlinear patterns, featuring a pronounced rise until age 60, followed by either stabilization or decline. Furthermore, our analysis uncovered that among the 299 metabolites linked to ageing, 297 of them exhibited interactions with sex (Extended Data Fig. 6a–f and Supplementary Table 24).

In addition, to quantify the metabolomic changes occurring during ageing, the differential expression-sliding window analysis (DE-SWAN)^23^ identified two crests in metabolite expressions at ages 46 and 64 (Fig. 5h–j and Supplementary Table 25). Notably, although the top 15 age-related metabolites differed at ages 46 and 64, there was an obvious overlap (*n* = 161; Fig. 5i). Metabolites in cluster 1, for example, the ratio of omega-6 fatty acids to omega-3 fatty acids (omega-6/omega-3) and the histidine-to-citrate ratio (His/Citrate), exhibited a consistent decreasing trend at both crests, reflecting their age-associated decline. In contrast, most of the top age-related metabolites were from clusters 3, 4 and 5, and they largely followed converse directions that increased at age 46 and decreased at age 64 (Fig. 5j and Supplementary Table 26). These findings imply that ageing is a dynamic process characterized by waves of changes in plasma metabolites.

### Machine-learning-based MetRS facilitates disease discrimination

**Fig. 6:**
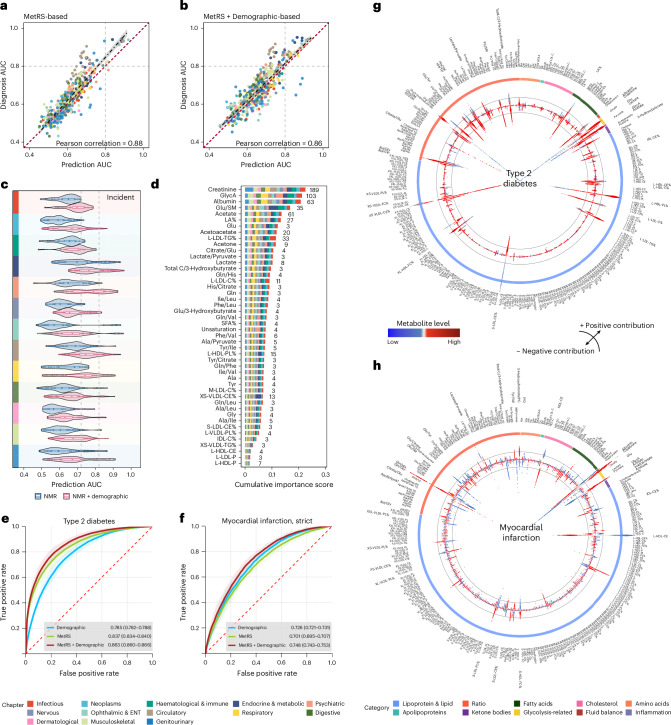
Machine-learning-based MetRS facilitates disease prediction. **a**,**b**, Correlation between AUC values of 472 shared endpoints in incident diseases based on the MetRS model (**a**) and the MetRS + Demographic model (**b**). MetRS leveraged the top 30 metabolites based on the ranked importance scores. Each dot represents a distinct disease endpoint. The dashed regression line represents the best-fit linear model between predicted and observed AUCs. The shaded band around the regression line denotes the 95% CI. **c**, Discriminative performances of metabolites in disease prediction (AUCs) based on two models: MetRS and MetRS + Demographic. Violin plots represent the distributions of AUC values of specific disease chapters (the analysed diseases within each chapter are provided in Supplementary Table 4). The violin plot ranges represent the minimum to maximum values, box ranges (quartiles) and median values. **d**, Stacked bar plot showing metabolites’ cumulative importance represented by normalized information gain, derived based on light gradient-boosting machine (LightGBM) models. Numbers indicate the count of diseases where each metabolite achieved top predictive importance. Metabolites served as the top contributor for at least three diseases included in this chart. **e**,**f**, ROC curves for predicting T2D (**e**) and myocardial infarction (**f**) using the Demographic model alone, MetRS alone and MetRS + Demographic models. ROC curves are shown with shaded bands indicating 95% CIs. AUC values are reported as mean ± 95% CIs. **g**,**h**, Circular SHAP value plots illustrating metabolites’ contributions to T2D (**g**) and myocardial infarction (**h**) predictions. The coloured outer ring represents metabolite groups. The width of the bars located on the inner ring indicates the extent of metabolites’ contribution to disease prediction, with wider bars reflecting a greater contribution. The top 30 important metabolites are marked in bold. The colour of the bar represents the magnitude of metabolites, ranging from low (blue) to high (red), as depicted in the colour bar between **g** and **h**. Deviations towards the centre and periphery signify negative (protective) and positive (risk) contributions, respectively. Colour panels are shared in **a**–**d**, representing disease chapters, and in **g** and **h**, representing metabolite categories, shown at the bottom. BMI, body mass index; TDI, Townsend deprivation index. Source data

To investigate the discriminative capability of the metabolome, we leveraged machine learning to establish MetRS as metabolic risk representations for prevalent and incident diseases. The MetRS exhibited comparable performance for both, with a Pearson correlation of 0.88 between the area under the receiver operating characteristic (ROC) curves (AUC). The MetRS for prevalent diseases slightly outperformed that for incident ones, while incorporating demographic data further aligned the performance (Fig. 6a,b).

For disease prediction, MetRS exhibited moderate to excellent discriminative performance with AUCs surpassing 0.7 for 100 (11.6%) incident diseases, among which 28 of them witnessed good AUCs exceeding 0.80, especially in endocrine and metabolic diseases (*n* = 13). Of note, the MetRS excellently predicted future diabetic complications, for example, diabetic maculopathy (AUC = 0.921 (95% CI, 0.914–0.928)), diabetic kidney failure (AUC = 0.919 (0.906–0.930)) and type 2 diabetes (T2D) with peripheral circulatory complications (AUC = 0.913 (0.898–0.926)). By integrating MetRS with demographic information, prediction performance demonstrated improvement that 81 diseases obtained AUCs that surpassed 0.8. In comparison to demographic information, MetRS alone exhibited better performance (DeLong *P* < 0.05) in 61 (7.1%) diseases; moreover, MetRS demonstrated added values on top of demographic information (AUC of MetRS + Demographic greater than of demographics alone, DeLong *P* < 0.05) in 527 (61.4%) diseases. The added values of MetRS largely existed in disease categories of digestive, circulatory and endocrine/metabolic diseases (Fig. 6c and Supplementary Table 29).

**Extended Data Fig. 7:**
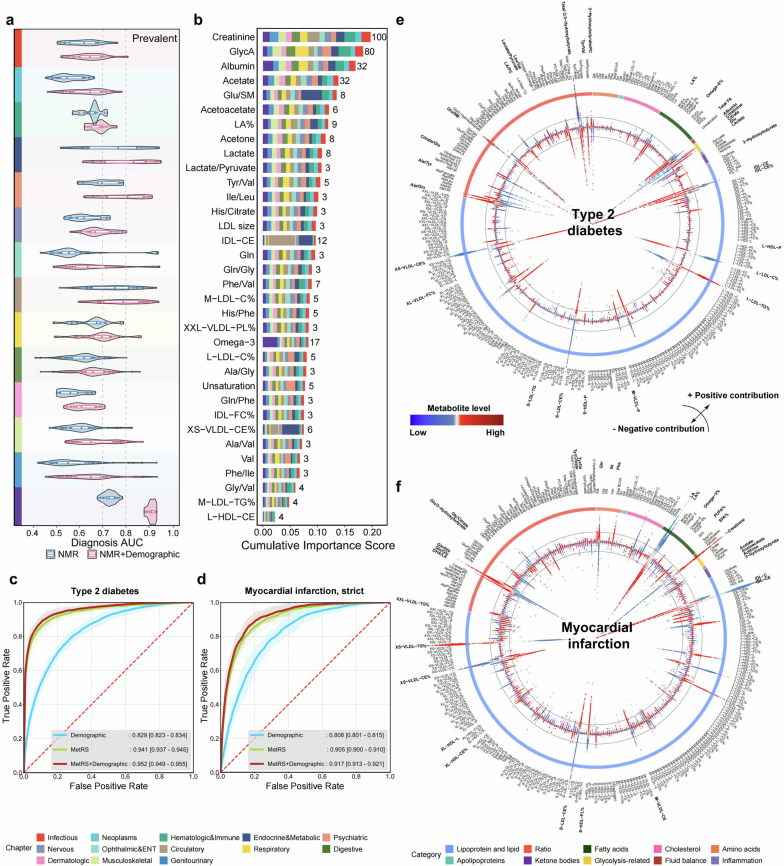
Metabolomic Risk Score facilitates prevalent diseases classification. **(a)** Discriminative performance (quantified by AUCs) of metabolites in classifying prevalent diseases based on two models: MetRS and MetRS+Demographic. MetRS leveraged top 30 metabolites based on the ranked importance scores. Violin plots represent the distributions of AUC values of specific disease chapters (the analyzed disease within each chapter are provided in Supplementary Table 3). The violin plot ranges represent the Min-Max, box ranges (quartiles), and median values. **(b)** Stacked bar chart of metabolite roles in classifications, colored by disease chapter. Numbers adjacent to the bars represent the number of diseases in which the importance of this metabolite ranked first. Metabolites served as the top contributor for at least 3 diseases were included in this chart. (**c-d**) ROC curves for classifying prevalent **(c)** type 2 diabetes and **(d)** myocardial infarction using Demographic alone, MetRS alone and MetRS+Demographic models. ROC curves are shown with shaded bands indicating 95% confidence intervals. AUC values are reported as mean±95% confidence intervals. (**e, f**) Circular SHAP value plots illustrating metabolic contributions to classify prevalent type 2 diabetes **(e)** and myocardial infarction **(f)**. The colored outer ring represents metabolite groups. The width of the bars located on the inner ring indicates the extent of metabolites’ contribution to the classification, with wider bars reflecting a greater contribution. Top 30 important metabolites were marked in boldface. The color of the bar represents the magnitude of metabolites, ranging from low (blue) to high (red), as depicted in the color bar between **(e)** and **(f)**. Deviations toward the center and periphery signify negative (protective) and positive (risk) contributions, respectively. Color panels are shared in (**a-b**), representing disease chapters, and (**c-d**), representing metabolite categories, shown at the bottom. AUC, the area under the Receiver-Operating-Characteristic (ROC) curve; MetRS, Metabolite risk score. Source data

For the classification of prevalent diseases, 35 MetRS yielded a good performance of AUC > 0.8, particularly in circulatory (*n* = 14) and endocrine and metabolic (*n* = 10) diseases. The MetRS witnessed excellent diagnosis for type 1 diabetes (T1D; AUC = 0.944), T2D (AUC = 0.941), diabetic maculopathy (AUC = 0.940) and chronic kidney disease (CKD; AUC = 0.933). Furthermore, in combined MetRS with demographics, 94 (17.8%) diseases showed AUC > 0.8. In comparison to demographic information, MetRS alone showed better performance in 17.8% (*n* = 94) of diseases; moreover, MetRS illustrated added values to demographics in 61.3% (*n* = 323) of diseases, which were mainly found in digestive and circulatory diseases (Extended Data Fig. 7a and Supplementary Table 27).

**Extended Data Fig. 8:**
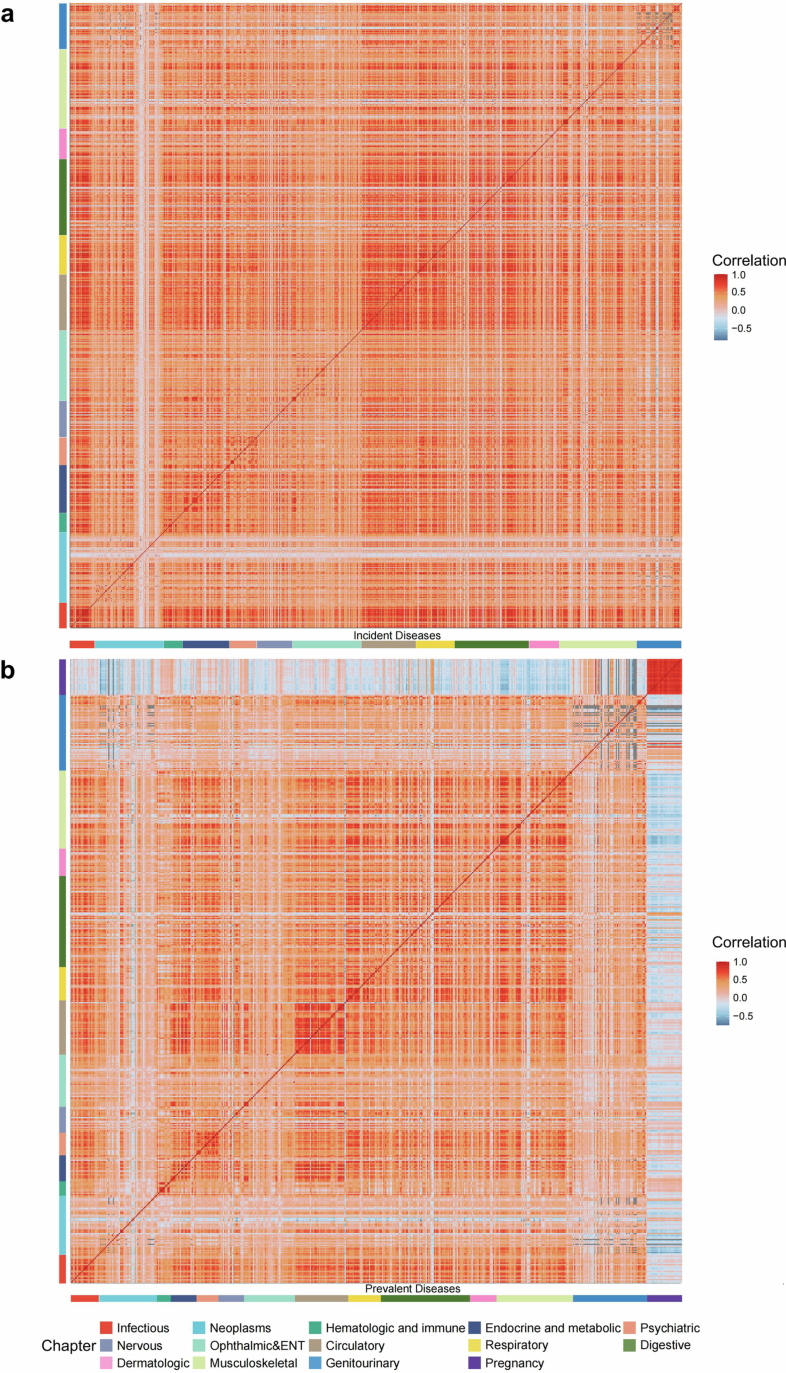
MetRS correlation heatmap between (a) incident and (b) prevalent disease pairs. Heatmaps showing pairwise correlations of metabolite risk scores (MetRS) across **(a)** incident diseases and **(b)** prevalent diseases. Each cell represents the correlation coefficient between MetRS profiles for two diseases. The color scale indicates the direction and magnitude of the correlation. Diseases are organized and color-coded according to their respective chapters, as indicated by the color bars along the axes. Source data

To identify critical metabolic markers of disease discrimination, we sorted the metabolites based on their importance. Of note, creatinine, GlycA, albumin and acetate were top-ranked markers in both prevalent disease classification and incident disease prediction. Specifically, creatinine and GlycA emerged as the most influential markers among 189 (22.0%) and 103 (12.0%) diseases in predictions (Fig. 6d and Supplementary Table 30), while they were dominant in 100 (19.0%) and 80 (15.2%) diseases for classification (Extended Data Fig. 7b and Supplementary Table 28). The MetRS demonstrates obvious patterns of correlation within and between disease groups (Extended Data Fig. 8), which aligns with previous findings, indicating potential shared underlying metabolic pathways^9,24^.

The prediction of T2D and myocardial infarction obtained AUCs of 0.863 (0.860–0.866) and 0.748 (0.743–0.753), respectively (Fig. 6e,f), with classification both showing much better performance of 0.952 (0.949–0.955) and 0.917 (0.913–0.921; Extended Data Fig. 7c,d). As depicted in Fig. 6g, SHapley Additive exPlanations (SHAP) values^25^ revealed an elevated ratio of glucose to sphingomyelins (Glu/SM) and glucose levels increased future T2D risk, whereas a decreased ratio of cholesteryl esters to total lipids in the very small VLDL percentage (XS-VLDL-CE%) suggests reduced risk. Increasing creatinine and the glutamine/glycine ratio (Gln/Gly) showed a higher risk of a future myocardial infarction, while higher cholesteryl esters in large HDL (L-HDL-CE) and albumin levels conferred protection (Fig. 6h). Consistent findings were found in prevalent diseases (Extended Data Fig. 7e,f).

### Mendelian randomization and colocalization prioritize causal metabolites

To further elucidate potentially causal relationships between metabolites and diseases and identify potential therapeutic targets, Mendelian randomization and colocalization analyses were performed. We identified 7,570 potentially causal metabolite–disease associations (*q* value < 0.05) in forward Mendelian randomization analyses (Supplementary Table 31). The number of identified associations per metabolite ranged from 1 to 101. Across 11 disease categories, circulatory (*n* = 1,914) and endocrine and metabolic (*n* = 1,250) categories had the most associations. In sensitivity analyses, 6,196 (81.9%) metabolite–disease associations remained statistically meaningful after excluding single nucleotide polymorphisms (SNPs) linked to dietary intake (Supplementary Table 32). When a stricter instrumental variables selection was applied (Methods), the number of associations was 544 (Supplementary Table 33). Of these, 454 associations were consistently statistically meaningful across both the primary and sensitivity analyses (Supplementary Table 34).

**Fig. 7:**
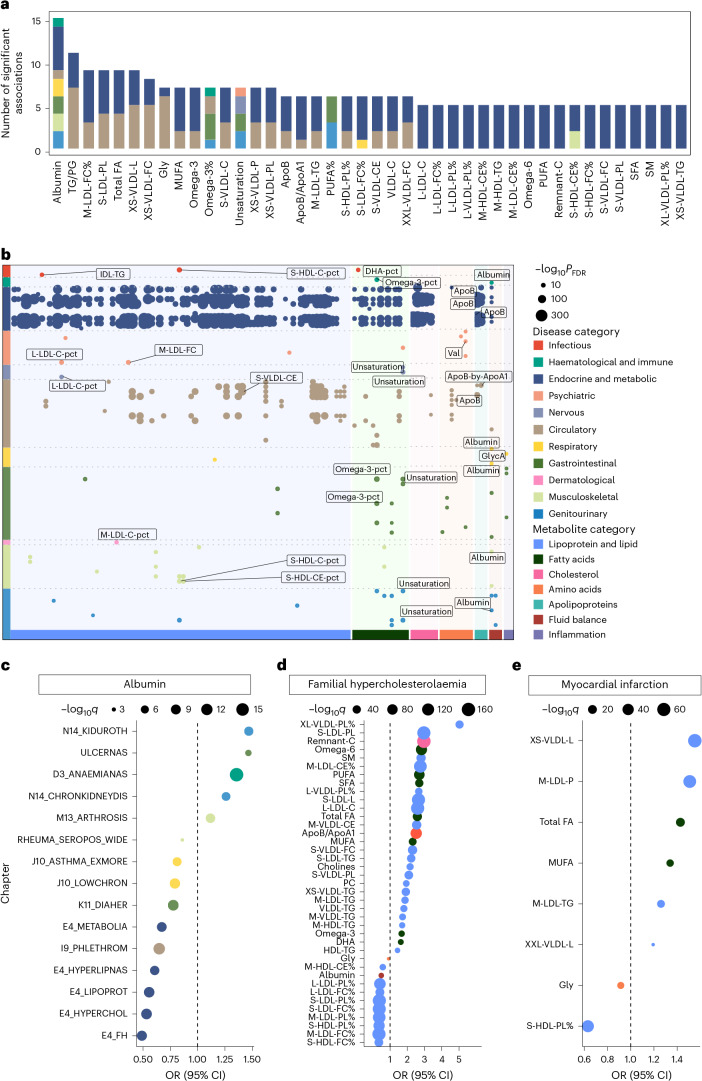
Mendelian randomization reveals candidate causal metabolites across disease categories. **a**, Stacked bar plot showing potentially causal metabolites most correlated with diseases, coloured by disease chapter. Metabolite–disease pairs that were consistently statistically meaningful across both the primary and sensitivity analyses were included. **b**, Summary of metabolite–disease causal relationships that were consistently statistically significant across both the primary and sensitivity analyses. The *x* axis represents metabolite categories, the *y* axis lists disease chapters, and the dots represent the statistically meaningful causal relationships between metabolites and diseases. Colours represent disease chapters, and annotations indicate the top three diseases (by *P* value) in each disease chapter. The dot size reflects significance level (−log_10_*q*), with larger dots indicating higher significance. FDR, false discovery rate. **c**, Mendelian randomization results for albumin and various diseases. The *x* axis shows the OR value, with diseases coloured by chapter. The size of the dots represents the significance level (−log_10_*q*), where *q* values were derived from two-sided tests and adjusted for multiple comparisons using the FDR method. Full disease names corresponding to each abbreviation are provided in Supplementary Table 4. **d**,**e**, Results of Mendelian randomization analysis for various metabolites and familial hypercholesterolaemia (**d**) and myocardial infarction (**e**). The *x* axis represents the OR value, with metabolites listed along the *y* axis. Dot size indicates significance. *P* values were computed using two-sided tests and corrected for multiple testing using the FDR method. Source data

Among the 454 shared associations, the number of identified associations per metabolite ranged from 1 to 15 (Fig. 7a). Among the 11 disease categories, endocrine and metabolic (*n* = 295) and circulatory categories (*n* = 87) had the highest numbers of associations (Fig. 7b). Albumin was associated with most diseases (*n* = 15), positively linking to a higher incidence of ulcerative colitis, certain types of anaemias and CKD (Fig. 7c). Moreover, the ratio of phospholipids to total lipids in the very large VLDL percentage (XL-VLDL-PL%) exhibited the largest effect size in increasing the risk of familial hypercholesterolaemia (odds ratio (OR) = 5.03 (3.97–6.38), *q* value = 7.39 × 10^−39^), while the ratio of free cholesterol to total lipids in the small HDL percentage (S-HDL-FC%) was the most evident protective metabolite (OR = 0.35 (0.30–0.40), *q* value = 4.28 × 10^−48^; Fig. 7d). Furthermore, total lipids in very small VLDL (XS-VLDL-L) exhibited the strongest association with increased risk of myocardial infarction (OR = 1.55 (1.48–1.63), *q* value = 1.56 × 10^−64^), while the ratio of phospholipids to total lipids in the small HDL percentage (S-HDL-PL%; OR = 0.63 (0.59–0.67), *q* value = 7.03 × 10^−48^) exhibited the most notable protective effect (Fig. 7e).

**Extended Data Fig. 9:**
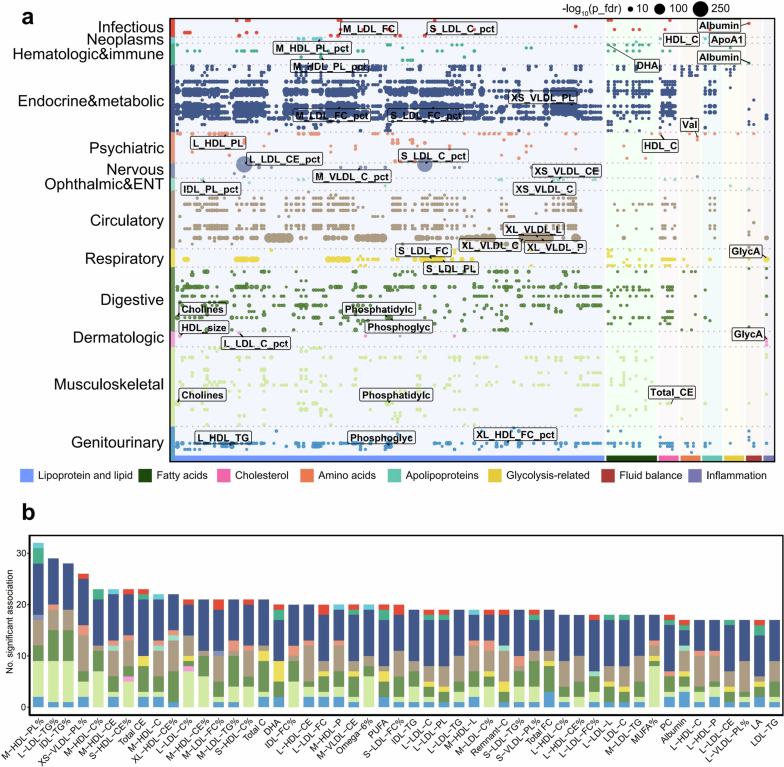
Summary of potential consequential metabolites. **(a)** Summary of statistically significant metabolite-disease consequential relationships in reverse Mendelian randomization analysis. The x-axis represents metabolite categories, the y-axis lists disease chapters, and the dots represent the causal relationships between metabolites and diseases. Colors represent disease chapters, and annotations indicate the top three diseases (by P-value) in each disease chapter, the dot size reflects significance level (−log10(P-*fdr*)), with larger dots indicating higher significance. **(b)** Stacked bar plot of potential metabolites that may result from certain diseases. Only metabolites with associations greater than 20 diseases were plotted. Colors are shared with **(a)** represent disease chapters. Source data

**Extended Data Fig. 10:**
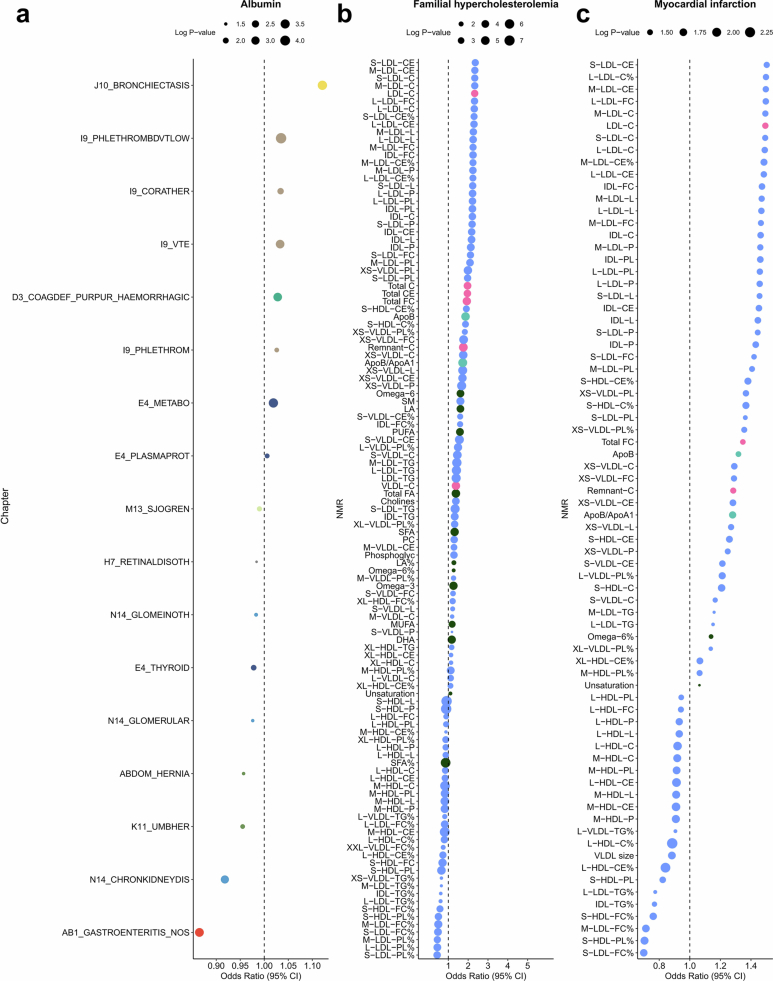
Statistically significant reverse mendelian randomization results linking metabolites and diseases. **(a)** Statistically significant results of reverse Mendelian randomization analysis for Albumin and various diseases, colored by disease chapter. The x-axis shows the odds ratios. The size of the dots represents the significance level (−log10(P*fdr*)), where P values were derived from two-sided tests and adjusted for multiple comparisons using the FDR method. Full disease names corresponding to each abbreviation are provided in Supplementary Table 4. (**b-c**) Statistically significant results of reverse Mendelian randomization analysis for metabolites and familial hypercholesterolemia **(b)** and myocardial infarction **(c)**. The x-axis represents the odds ratios, with metabolites listed along the y-axis. Dot size indicates significance and is colored by metabolite categories. P values were computed using two-sided tests, and corrected for multiple testing using the FDR procedure. Source data

The investigation into potential causality also provided clues that certain diseases may influence metabolite levels. Reverse Mendelian randomization analysis identified 2,679 disease–metabolite pairs, with triglycerides to total lipids in the large LDL percentage (L-LDL-TG%) associated with the most diseases, potentially influenced by genetic liability to 175 diseases across 12 categories (Extended Data Figs. 9 and 10 and Supplementary Table 35). Interestingly, albumin levels also showed reciprocal causal associations with genetic liability to the risk of ulcerative colitis, certain types of anaemias and CKD.

**Fig. 8:**
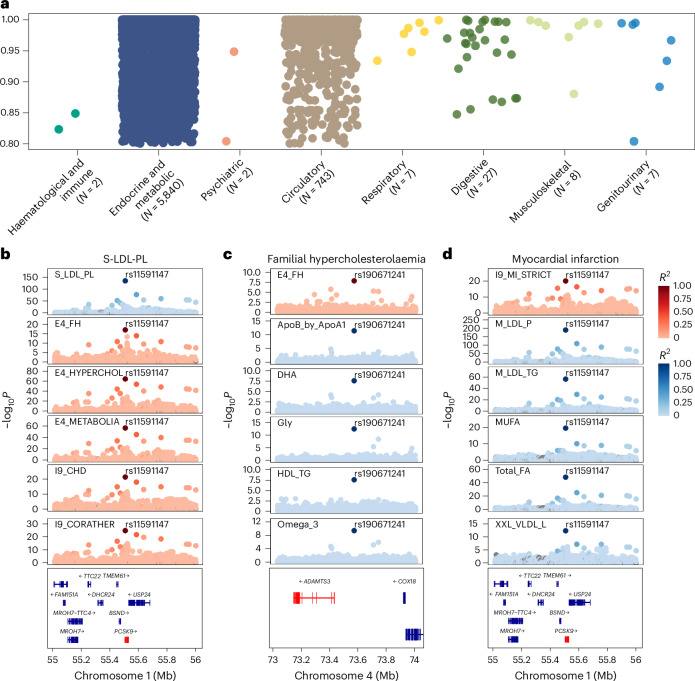
Genetic colocalization reveals pleiotropic variants linking metabolites and diseases. **a**, Colocalization analysis of metabolite–disease associations. The *y* axis lists the disease categories, and the number of associations for each category is indicated. The *x* axis represents the posterior probability of colocalization, ranging from 0.80 to 1.00. Dots represent colocalized metabolite–disease associations, coloured by disease category. **b**–**d**, Colocalization of genetic variants with metabolites and diseases. **b**, Statistically meaningful colocalization signals between the genetic variant rs11591147 and S-LDL-PL with multiple diseases. **c**, Colocalization relationships for familial hypercholesterolaemia at the genetic variant rs190671241 with several metabolites. **d**, Colocalization relationships for myocardial infarction with several lipoprotein lipids at the genetic variant rs11591147. Each plot shows the −log_10_*P* values along the chromosome position; the colour coding in all panels represents the linkage disequilibrium (*R*^2^) with the top variant. Source data

For a potentially causal association implicated in the Mendelian randomization analysis, we further conducted colocalization analysis to investigate the shared genetic determinants. Among the 454 metabolite–disease associations, 402 had profiles supportive of colocalization (PP.H4 > 0.8; Supplementary Table 36). Evidence of shared genetic architecture was identified across a range of disease categories, with the endocrine metabolic and circulatory categories exhibiting the highest number of colocalized signals (Fig. 8a). Among all the loci, rs2954021 mediated the most metabolite–disease associations. For instance, the ratio of triglycerides to phosphoglycerides demonstrated colocalization evidence with several cardiovascular and metabolic diseases at rs964184. We also observed colocalization evidence between non-alcoholic fatty liver disease and alanine content at rs964184.

Among all the metabolites, phospholipids in small LDL (S-LDL-PL) had the most colocalized signals, with 176 shared variants associated with nine unique diseases. For example, rs11591147 may be responsible for the association between S-LDL-PL and multiple diseases, including familial hypercholesterolaemia, pure hypercholesterolaemia, major coronary heart disease events, coronary atherosclerosis, ischaemic heart disease and angina pectoris (Fig. 8b). Notably, rs11591147 in *PCSK9* was associated with LDL level and coronary artery disease risk across diverse populations^26–28^. Our findings further provided compelling evidence that rs11591147 is likely the causal variant underlying the widespread associations between metabolites and various diseases. In addition, familial hypercholesterolaemia exhibited a colocalization relationship with multiple lipoproteins and lipids, fatty acids, along with glycine, remnant cholesterol, albumin, and a ratio related to apolipoprotein at rs190671241. Myocardial infarction showed strong evidence of colocalization with several lipoprotein lipid concentration indicators related to LDL and VLDL at rs11591147 (Fig. 8c,d).

We conducted sensitivity analysis for Mendelian randomization and colocalization analysis using the UKB-derived metabolite genome-wide association studies (GWAS) dataset. Detailed results are shown in Supplementary Tables 37–39.

### Interactive web tool enables in-depth exploration of metabolome–phenome atlas

To facilitate an in-depth exploration of the detailed results, an interactive web tool (https://metabolome-phenome-atlas.com/) was structured into four sections: (1) epidemiological association (disease-/trait-/metabolite-wide association analysis), (2) metabolite variation assessments at different times and ages, (3) genomic association (Mendelian randomization and colocalization analysis) and (4) disease discrimination (machine-learning classification analysis). The web tool was established under the CC BY-NC-ND 4.0 license for noncommercial use only.

## Discussion

This study introduced the largest human metabolome–phenome atlas, encompassing a comprehensive collection of health and disease phenotypes in 274,241 individuals, with a follow-up duration of 14.9 years. By evaluating over 1.4 million potential associations, the atlas uncovered 52,836 metabolite–disease associations and 73,639 metabolite–trait associations. The atlas mapped metabolite variations assessed at different times before diagnosis, revealing key time points of metabolic changes over a decade preceding onset. Machine-learning-based MetRS demonstrated favourable performance (AUC > 0.8) for discriminating 81 incident and 94 prevalent diseases. In search of promising therapeutic targets, we identified 454 potentially causal metabolite–disease associations, among which 402 share common genetic determinants. Taken together, the open-access atlas provides a comprehensive panorama of the metabolome–phenome, advancing the research community by improving the understanding of disease pathophysiology, and guiding the development of biomarkers for enhanced diagnostic, predictive and treatment strategies.

This study represents a groundbreaking systematic integration of the metabolome with comprehensive human health and disease, enabling the simultaneous identification of shared and specific metabolic alterations across diverse phenotypes. Our findings successfully validated more than half of the metabolite–disease associations identified in prior research based on the first round of released NMR data^17^. Moreover, we have identified over 7,000 metabolic ratios linked to various diseases, along with more than 60,000 associations between traits and metabolites. New findings include the association between higher levels of omega-6% and an elevated risk of gout, the positive correlations of medium HDL, and ApoA1, with alcohol-related mental and behavioural disorders, and the positive association between alanine/glutamine and T2D. These metabolite–disease associations highlight underlying mechanisms that warrant further investigation. Of note, while our cross-sectional analysis revealed numerous disease–metabolite associations, we acknowledge that these relationships require different interpretations depending on the disease type. For chronic conditions like T1D, these associations likely reflect both ongoing disease processes and treatment effects. For acute conditions, the metabolic alterations we observed may represent either predisposing factors that increase disease susceptibility or persistent metabolic changes following acute episodes, which could be valuable for understanding disease risk and recovery patterns^29^. Therefore, these metabolic signatures should not be viewed as diagnostic indicators.

A considerable clinical advancement from our study is that we utilized machine learning to establish the MetRS as a risk metric to identify cost-effective biomarker panels for evaluating disease risk. While a recent study also explored metabolomic risk scores, focusing on predictive performance for a set of 12 diseases based on NMR data^24^, it was limited by the number of outcomes considered and the analytical strategies used. Our research addresses this gap by integrating metabolomics data with a comprehensive spectrum of human diseases and health phenotypes. Moreover, our study adopted a multifaceted analytical framework to systematically map metabolite–disease associations, characterize metabolite variation, estimate MetRS, reveal causal relationships and genetic colocalization patterns. Additionally, this atlas ranked plasma metabolites based on their importance, highlighting that a few key metabolites could enhance risk stratification and disease prevention^9^. MetRS offered favourable diagnostic and predictive performance, particularly in T2D^30–32^, CKD^11,33^ and myocardial infarction^34^, aligning with previous studies. Previously underestimated, the highest ranking of GlycA and creatinine across all diseases demonstrated their critical diagnostic and predictive values, including GlycA, a strong predictor for lung cancer and Graves’ disease, while creatinine effectively predicted gout and cardiac arrest. This approach bridged a knowledge gap, emphasizing metabolites’ broad applicability as a universal biomarker and their potential for advancing precision medicine.

Because metabolomics is particularly crucial for pharmacological studies in search of promising pharmaceutical interventions^35^, Mendelian randomization and colocalization analysis provide a road map to identify potential high-value therapeutic targets^36^. Our atlas advances findings from our companion genetic investigation^37^, which systematically unveils the intricate genetic architecture underlying plasma metabolic traits. Together, these two complementary studies integrate genetic variation, metabolic profiles and clinical outcomes, providing a comprehensive framework for uncovering mechanisms of disease and enhancing disease predictive and treatment strategies. Furthermore, covering a comprehensive spectrum of diseases offers opportunities to evaluate the safety of possible targets. We rigorously controlled for the impact of diet on metabolites, and 81.9% of the causal metabolite–disease pairs remained statistically meaningful. Most potentially causal metabolite–disease pairs were found in metabolic and cardiovascular diseases, with the well-known bidirectional causal relationship between albumin level and CKD^38,39^. Different medications targeting this metabolite have been and continue to be developed, demonstrating the remarkable promise of potentially causal metabolites in the treatment of diseases. Moreover, as indicated by the causal relationship between albumin and metabolic diseases revealed in this atlas, while lowering albumin levels may decrease CKD events, it might also increase the risk of metabolic diseases. These findings suggest this atlas’s value in identifying novel and safe targets for future pharmaceutical interventions towards a wide range of human diseases.

This study opens several promising avenues for future research. The atlas revealed numerous remarkable and unexplored associations, requiring further fundamental experiments in animal models to decipher the complex metabolic pathways involved. To ensure the robustness and generalizability of our findings, we aspire to validate them in international consortia and multicentre prospective external cohorts. Additionally, comprehensive studies across the gene–transcript–protein–metabolite continuum in humans, especially focusing on tissue-specific and organ-specific levels, can lead to new therapeutic targets. Finally, future research should incorporate small-molecule metabolites into disease classification to enhance precise diagnosis and personalized therapeutic strategies.

While the study provides extensive insights into the metabolomic underpinnings of health and disease, some limitations should be noted when interpreting the results. First, the findings aspire to be further validated through independent external datasets; however, cohorts with large-scale populations, deep phenotypes and long-term follow-up are scarce. To alleviate this issue, we performed two internal replication analyses and found potential heterogeneity in the non-white population. It is important to note that replication within a single cohort may still suffer from similar confounding biases, as confounding structures are likely to be the same across different subgroups. The poorer replication performance observed in the non-white population may, in part, be due to differences in confounding structures related to demographic factors^40^. Additionally, there is a substantial difference in the sample sizes between the replication cohorts (tenfold), which could also contribute to the observed differences in validation rates. Secondly, the ^1^H-NMR technology assesses essential metabolites; however, more metabolites can be detected through additional techniques such as mass spectrometry. Additionally, we have incorporated a biological knowledge-based approach^36,41^ to enrich diverse metabolic ratios. Further, NMR-based metabolites offer significant advantages, including lower costs, greater feasibility for large-scale studies and more effortless clinical utility^42^. Furthermore, disease status in our study was determined using ICD-10 codes, which, while widely used in epidemiological studies, have inherent limitations. One major limitation is that ICD-10 codes may not fully capture all stages of chronic progressive diseases^43^. For example, diseases with a slow and variable progression may only be identified in later stages using ICD-10 coding, potentially excluding individuals in the early or subclinical phases. This inherent limitation means that our findings may not capture the full spectrum of disease progression, especially for conditions with a more gradual onset. Lastly, while we attempted to minimize the impact of diet-related genetic variation by excluding SNPs associated with dietary intake, the complex interplay between diet, metabolic pathways and disease makes it challenging to fully isolate dietary effects.

Moreover, while we accounted for sex and ethnic background in the DE-SWAN analysis, we acknowledge that other factors, such as lifestyle and living environment, may also contribute to the observed waves of change. Future studies should aim to uncover the underlying biology of these ageing waves and consider additional confounding factors to enhance our understanding of the ageing process. Finally, as our Mendelian randomization analyses were based on European populations, further studies are needed to establish the generalizability in diverse ancestral groups^44^. The highly correlated nature of metabolites and pleiotropic effects of genetic variants also pose inherent challenges for Mendelian randomization analysis, despite our exclusion of highly pleiotropic variants and sensitivity analyses. Although multivariable Mendelian randomization has the potential to address these issues^44–47^, simultaneously analysing hundreds of metabolites remains computationally prohibitive with current methods. Further methodological developments are needed to better handle high-dimensional metabolomics data.

In summary, the open-access human metabolome–phenome atlas is poised to deepen our understanding of human health and disease by unveiling the metabolic complexities in human biology. While this ambitious study offers comprehensive, actionable insights into the potential of metabolites for disease diagnosis, prediction and treatment, these insights necessitate careful research and thoughtful application. To guide these efforts, we offer a human metabolome–phenome atlas to inspire personalized healthcare and precision medicine.

## Methods

### Study population

This study adopted participants recruited from the UKB, a community-based cohort comprising adults aged between 40 and 69 years. Over 500,000 participants were recruited from 22 assessment centres across the United Kingdom between 2006 and 2010, and they were all registered with the UK National Health Service. This study included 274,241 participants who underwent metabolic profiling of their blood plasma samples collected during the baseline visits. The NMR metabolites were measured in two phases. We split the data into 148,974 individuals of white ancestry released in phase 2 (assessed between April 2020 and June 2022) as a derivation cohort, 111,826 individuals of white ancestry released in phase 1 (assessed between June 2019 and April 2020) as replication cohort 1 and 13,444 individuals of non-white ancestry from both phases as replication cohort 2 (Supplementary Table 1 and Extended Data Fig. 1a).

### Ethics

The study was conducted following the Declaration of Helsinki. Ethical approval was obtained from the North West Multi-centre Research Ethics Committee (MREC; https://www.ukbiobank.ac.uk/learn-more-about-uk-biobank/about-us/ethics/). All study participants provided informed consent. This study was approved by the UKB under application numbers 202239 and 19542.

### Plasma NMR metabolic profiling

Blood samples were collected in ethylene diamine tetraacetic acid vacutainers and promptly centrifuged at 2,500*g* for 10 min at 4 °C to separate the plasma and before being preserved at −80 °C for storage. The blood sample handling and storage protocol has been previously described^48^. The metabolic profiling of ethylene diamine tetraacetic acid samples was performed through the Nightingale Health NMR biomarker platform using high-throughput NMR spectroscopy^49^. Detailed descriptions of sample preparation, metabolite measurements and quality controls can be found at https://biobank.ndph.ox.ac.uk/showcase/ukb/docs/NMR_companion_phase2.pdf.

The 313 metabolic measures were categorized according to the UKB NMR classification, of which 64 were derived ratios calculated through a knowledge-based integration of existing metabolites^36,41^, and 249 were quantified through NMR and classified into 10 categories, including amino acids, apolipoproteins, cholesterol, fatty acids, fluid balance, glycolysis-related metabolites, inflammation, ketone bodies, lipoproteins and lipids. The 249 NMR-quantified measures include pre-existing ratios (for example, ApoB/ApoA1, omega-6/omega-3, polyunsaturated fatty acids/monounsaturated fatty acids, the ratio of triglycerides to phosphoglycerides) derived before our analysis. For consistency, these pre-existing ratios and additional knowledge-based-derived ratios are unified under the category of ‘ratio’ in this analysis (Supplementary Table 2). The raw metabolic values were preprocessed by removing outliers, defined as any data outside four interquartile ranges from the median. Next, natural-log transformation and *z*-transformation were subsequently performed before further analysis. To account for the high intercorrelation among metabolites, we conducted a comprehensive pairwise correlation analysis across all metabolites. Spearman correlation coefficients were calculated to quantify the relationships between metabolite levels.

### Disease outcome definition

The diagnostic data in the UKB were obtained from UK Hospital Episode Statistics data. Disease endpoints were defined based on the first occurrence of the three-character ICD-10 code using the hospital inpatient records (fields 41270 and 41280). Diseases were categorized into prevalent and incident, defined as events that were present before or after participants’ baseline visits, at the same time when blood samples were collected. Follow-up began from the date of attendance to the assessment centres (baseline) to the earliest recorded date of diagnosis, the date of death or the final available date from hospital inpatient records (November 2023), whichever occurred first.

We adopted disease endpoint codes and quality-control guidelines from FinnGen (https://www.finngen.fi/), incorporating predefined criteria based on sex-specific, age-specific and disease-specific control exclusions. The ICD-10 codes classified disease outcomes into 14 chapters, including infectious, neoplasms, haematological and immune, endocrine and metabolic, psychiatric, nervous, ophthalmic, circulatory, respiratory, digestive, dermatological, musculoskeletal, genitourinary and pregnancy. Analysed diseases were subjected to quality control under guidelines provided by FinnGen, including predefined conditions based on sex or age and inclusion and exclusion criteria for specific diseases (Supplementary Tables 3 and 4).

For incident diseases, those with prevalent diagnosis records were excluded, and for prevalent diseases, those who developed the disease during the follow-up were also eliminated. In another saying, healthy controls for each disease were defined as individuals who neither had a prevalent diagnosis nor developed that specific disease throughout the past or follow-up period. Besides, diseases with events that occurred in less than 300 individuals (≈0.1% of the total population) were excluded to ensure sufficient analysable events. Overall, this study included 527 prevalent and 859 incident disease outcomes (Supplementary Tables 3 and 4).

### Health-related and imaging trait definitions

To gain deeper insights into the impact of metabolites on human health and to elucidate potentially causal pathways linking metabolites to diseases, we accessed data on health-related traits and multi-organ imaging traits from the UKB, which has collected health-related information from participants’ baseline visits and imaging data at the second follow-up. In total, we included 3,142 health-related and imaging traits. Trait variables were categorized into three data types: continuous (*n* = 2,408), ordered categorical (*n* = 505) and binary (*n* = 267). Health-related traits with sample sizes smaller than 10,000 were excluded. For binary traits, a minimum of 50 cases or controls were required for inclusion. Details of included traits and characteristics are provided in Supplementary Tables 5 and 6.

We incorporated a comprehensive array of 991 health-related traits, which were organized into 10 distinct categories corresponding to secondary or tertiary UKB paths: mental health (*n* = 235), health and medical history (*n* = 201), diet and food preference (*n* = 174), eye measures (*n* = 109), physical measures (*n* = 95), lifestyle factors (*n* = 77), blood and urine assays (*n* = 54), physical activities (*n* = 29), cognitive function (*n* = 16) and working and living environment (*n* = 1)^37^. The PEACOK R package^50^ was utilized to process UKB’s health-related traits.

Furthermore, our analysis comprised 2,151 imaging traits, spanning various modalities such as brain imaging (including T1 structural brain MRI, susceptibility-weighted brain MRI and diffusion brain MRI; *n* = 1978), as well as cardiovascular magnetic resonance (*n* = 129) and abdominal MRI traits (*n* = 44). Image acquisition followed predefined standard operating procedures using uniform equipment and staff training^51^. Specifically, multi-modal brain MRIs were conducted using Siemens 3T scanners following an extensive data processing and quality-control pipeline^52,53^. Cardiovascular magnetic resonance and abdominal scans were performed using Siemens 1.5T scanners with detailed protocols and processing described earlier^54–56^.

### Covariates

Covariates were selected based on (1) participants’ demographic information, including age at blood collection, sex and TDI; (2) potential factors associated with the plasma metabolites, including BMI, smoking status (never, former and current), medication of statin and fasting time before the blood collection. (3) We included eGFR as an additional covariate in the regression models. Missing values were less than 5% for all covariates; thus, simple imputation was adopted using sex-based mode and median for discrete and continuous variables, respectively. We formally tested interactions between metabolites and sex, as well as between metabolites and age, by including interaction terms in the regression models.

### Associations between metabolites and diseases

Regarding the date of disease diagnosis before and after blood collection, we performed cross-sectional and prospective analyses. Specifically, for cross-sectional analysis of prevalent diseases, logistic regressions were performed with the adjustment of covariates listed above, and the significance was determined using Bonferroni correction for multiple tests comparison, defined as *P* < 0.05/(313 × 527). For prospective analysis, Cox proportional-hazards regressions were used by adjusting the same set of covariates, and Bonferroni correction was applied using *P* < 0.05/(313 × 859). The logistic regression and Cox proportional-hazards regression were performed using the statsmodels (v0.13.1) and lifelines (v0.27.4) packages under Python.

### Associations between metabolites and traits

The associations between metabolites and traits were assessed using various regression models tailored to the specific types of traits. Linear regressions were adopted to test continuous and binary traits, while proportional odds logistic regression was applied for ordered categorical traits. Notably, during linear regressions, binary traits were treated as independent variables and metabolite levels as dependent variables. Inverse normal transformation was used for continuous traits to ensure data normality. All models adjusted the same set of covariates as aforementioned. Bonferroni correction was applied for multiple-testing comparison (*P* < 0.05/(313 × 3,142)). Regression models were conducted using the ‘lm’ and the ‘polr’ function from the MASS R package (v4.2.0).

### Nested case–control study of metabolite variation assessments at different times before disease diagnosis

We aimed to depict variations in metabolites assessed at different time windows preceding disease diagnosis for pre-identified metabolite–disease associations. A nested case–control study was performed^57–59^ (Extended Data Fig. 5a). Individuals diagnosed during the follow-up period were categorized as cases for each incident disease. To ensure comparability, each case was matched with five controls based on propensity scores, accounting for similar sex, age at baseline, self-reported ethnic background, BMI and TDI using the nearest-neighbour matching method. The time elapsed before clinical diagnosis (baseline date minus date of clinical diagnosis) was calculated for each case, and controls were assigned a ‘proxy’ time matching that of their respective matched case. Various timeframes preceding disease diagnosis (for example, year −15, year −12, year −10, up to disease onset) were considered. Within each timeframe, we computed the relative levels of metabolites in cases compared to controls using *z*-score transformation, with smoking, statin use and fasting time adjusted during regressions.

To determine the precise timeframe in which each metabolite begins to vary before disease onset, we used a backward screening strategy, hypothesizing that individuals would exhibit more obvious metabolite variation as they approached the disease onset. Specifically, we quantified the statistical difference between cases and nested controls for each timeframe using *P* values derived from Student’s *t*-tests. Subsequently, starting from the timeframe immediately preceding disease onset, we assessed the significance of each timeframe in succession. This process ceased when two consecutive timeframes became insignificant (*P* > 0.05), and we then designated the stopping timeframe as the point at which variation emerged before disease onset.

Furthermore, to identify diseases with similar metabolite variational patterns leading up to their onsets, hierarchical clustering was performed to group 859 incident diseases. Each disease was characterized using *z*-score-transformed metabolic measurements under sequential time frames preceding disease onset, represented as a 313 × 15 matrix (number of metabolites × number of timeframes). Hierarchical clustering was conducted based on the Ward linkage method, and the Silhouette method was used to determine the optimal number of clusters.

### Clustering metabolites at different ages of assessments

To estimate the variation of metabolites at different ages, plasma metabolite levels were *z*-scored with the adjustment of sex, self-reported ethnic background, TDI, BMI, smoking, statin use and fasting time. To group metabolites with similar variational patterns across different ages of individuals at baseline visits, hierarchical clustering was performed on fitted metabolite levels using LOESS regressions. The hierarchical clustering was conducted using the Ward linkage on a pairwise differences matrix defined by the Euclidian distance. The optimal number of clusters was chosen based on the Silhouette score.

### Quantification of metabolite variations at different ages of assessments using DE-SWAN

We proceeded with the DE‐SWAN algorithm to identify and quantify natural changes in the plasma metabolome during ageing^23^. DE‐SWAN iteratively used each age in the input vector as the centre of a window and compared the metabolite level of individuals in parcels below and above this window. With the window sliding in increments of 1 year from young to old, we can detect changes at stages of life and determine the sequential effects of ageing on metabolites with confounding factors adjusted. We binarized the age according to the parcels and used the following linear model to test for metabolite changes in waves throughout ageing as given by equation (1):

1 $${\rm{Metabolite}} \sim \alpha +{\beta }_{1}\times {\rm{age}}_{\frac{\rm{Low}}{\rm{High}}}+{\beta }_{2}\times {\rm{sex}}+{\beta }_{3}\times {\rm{ethnic}}\; {\rm{background}}\;+\varepsilon$$

where *ε* is the random error term of the model. For each window, adjusted *P* values were estimated using the Benjamini–Hochberg correction. Moreover, multiple parcel widths (2, 3, 4 and 5 years) and numerous *q*-value thresholds (0.0001, 0.001, 0.01, 0.05) were tested to assess the robustness and relevance of DE-SWAN results.

### MetRS estimates the risk for prevalent and incident diseases

The MetRS were respectively developed for each target outcome of prevalent and incident diseases. Specifically, for prevalent diseases, the MetRS was developed to quantify the risk of an individual previously diagnosed or currently experiencing a particular disease. For incident diseases, the MetRS was established to determine the risk of a baseline healthy individual developing a particular disease in the future.

The MetRS was developed by initially training a naive classifier of LightGBM^60^ with all 313 metabolites. Next, a metabolic importance score was calculated based on an inherent algorithm of information gain, which measured the metabolite’s discriminative capability. Thus, for each disease, all metabolites can be ranked based on such an importance score, and the top 30 (≈10%) ones were extracted as key predictors to establish the MetRS (the top 30 metabolites for each disease were reported in Supplementary Tables 28 and 30 for prevalent and incident diseases, respectively). After that, a LightGBM classifier with fine-tuned hyperparameters was developed using a preselected list of the top 30 metabolites, and a post-processor of isotonic regression was used to calibrate the predicted risks to the observed disease prevalence. The final output probabilities, ranging between 0 and 1, were taken as MetRS. In addition, to improve the discriminative accuracy, we developed an integrated model on top of the MetRS by further incorporating easily accessible demographic information, including participants’ age, sex, self-reported ethnic background, TDI, BMI and smoking status.

To transparentize the contribution of metabolites, we used SHAP^25^ to explore the metabolites’ contributions within machine-learning models. The SHAP values enable us to visually delineate metabolites’ importance and their predictive effects, either positive or negative, on the target outcomes. We calculated SHAP values when modelling with all 313 metabolites and normalized them across each metabolite. SHAP values were computed using shap (v0.41.0) in Python.

The machine-learning pipeline was performed in a tenfold cross-validation strategy using all 274,241 individuals. Participants were split into ten folds based on the geographical locations of their baseline attendance. During modelling training, nine folds of data (training set) were used for MetRS development, including metabolite importance ranking, model hyperparameter tuning and model training, and the rest fold (testing set) was kept untouched and merely used for model evaluation. Such a training scheme was iteratively performed until each data fold served as either a training or a testing set. Next, all MetRS data derived from the testing set were then aggregated, and a bootstrap strategy with 1,000 iterations was applied to report the median and 95% CIs of evaluation metrics. We reported the top 30 metabolites by averaging the selected ones under ten cross-validation iterations. Model development and evaluations were implemented through lightgbm (v3.3.2) and scikit-learn (v1.0.2) in Python.

### Two-sample Mendelian randomization

For statistically associated metabolite–disease pairs, we further conducted bidirectional Mendelian randomization analyses to determine the direction of causation. We focused specifically on incident disease cases to minimize potential confounding from reverse causation or treatment-related effects on metabolite levels. We accessed GWAS summary statistics for 368 diseases from FinnGen release 10 (https://www.finngen.fi/en/access_results/) and performed GWAS analysis for the remaining 107 diseases in a subset of 220,000 white British UKB participants (https://metabolome-phenome-atlas.com/). Detailed source information is provided in Supplementary Tables 31–35 under ‘GWAS data source’. The GWAS analyses were performed using generalized linear mixed models implemented through the Genome-wide Complex Trait Analysis^61^ (GCTA, v1.94.0). Metabolite GWAS summary statistics were derived from a genome-wide meta-analysis of 233 NMR circulating metabolic traits, involving up to 136,016 participants from 33 cohorts^44^. Among these, we included data for 223 metabolic traits that overlap with the metabolic profile analysed in our study.

For primary bidirectional Mendelian randomization analyses, instrumental variables were selected using the PLINK 2.0 clumping function (clump-kb 500, clump-r2 0.1, clump-p1 5 × 10^−8^), and the European 1,000 Genomes phase 3 dataset was used as the reference genome. Additionally, we performed two sensitivity analyses for forward Mendelian randomization: (1) To minimize the potential influence of diet-related genetic variation, we excluded SNPs associated with dietary intake, which were identified from a GWAS on dietary habits in the UKB^62^; (2) To minimize potential horizontal pleiotropy, we excluded genetic variants associated with more than five metabolites (*P* < 5 × 10^−8^)^44,45^.

For the main analyses, we used two methods: the Wald ratio method for cases with a single instrument, the inverse-variance weighted (IVW) method when multiple instruments were available. To ensure reliable causal inference, we implemented multiple robust Mendelian randomization methods as sensitivity analyses, specifically: MR-Egger, weighted median, simple mode and weighted mode, with results presented in the Supplementary Tables 32 and 34. We conducted heterogeneity assessments through both IVW and Egger-based tests, and pleiotropy was assessed by MR-Egger intercepts. *F*-statistics were calculated for all genetic instruments. For IVW analyses, we used a multiplicative random-effects model due to observed heterogeneity across SNPs, allowing us to account for potential over-dispersion and variant-specific heterogeneity. Missing data were handled through complete case analysis. Results with *q* value < 0.05 after FDR multiple-testing correction were considered statistically meaningful. Analyses were performed using the TwoSampleMR (v0.6.1) R package.

### Colocalization analysis

To investigate if shared variants were responsible for the potentially causal associations observed in the Mendelian randomization analysis, further colocalization analysis was performed. For each metabolite GWAS, lead SNPs were ordered based on their level of statistical significance. These SNPs were iteratively filtered to ensure that each was at least 1 Mb away from any variant with higher significance. For each lead SNP, we obtained GWAS summary statistics for metabolites and disease traits within 1 Mb as input. The prior probabilities were set to *P*_1_ = 1 × 10^−4^, *P*_2_ = 1 × 10^−4^ and *P*_12_ = 1 × 10^−5^, as recommended^63^. A posterior probability PP.H4 ≥ 0.8 generated by coloc was considered strong evidence for a shared causal variant affecting both metabolites and diseases. Analyses were performed using coloc (v5.2.3) R package.

### Sensitivity analyses for Mendelian randomization and colocalization analysis

We repeated the primary Mendelian randomization and colocalization analyses using this UKB-derived GWAS as a sensitivity analysis. For each metabolite, outliers outside four times the interquartile range were removed, followed by natural-log transformation. A total of 189,846 white British participants with both metabolomics and genomic data were included. GWAS analysis was conducted using an additive linear regression model implemented in PLINK2.0. The covariates included age, ethnicity, sex, fasting time, month of assessment, genotype measurement batch, the top 40 genotype principal components, age indicators by sex interactions and ethnicity by sex interactions. Metabolite GWAS summary statistics are available to access through our web tool.

### Statistics and reproducibility

No statistical method was used to predetermine sample size, and no data were excluded from the analyses. To further investigate any variations of the associations between metabolites and phenotypes exhibited across different individual characteristics, we performed subgroup analyses stratified by sex and age (<60 and ≥60 years). Notably, for subgroup analysis of sex, covariates of sex were removed, whereas all other covariates remained consistent for the age-stratified subgroups. Notably, the number of diseases for subgroups varied due to sex-specified diseases or insufficient cases. Specifically, for cross-sectional analysis, analysed diseases for female, male, middle-aged and older adult subgroups were 512, 443, 518 and 480, respectively; for prospective analysis, the numbers of diseases were 844, 820, 853 and 855, respectively. The significance defined by Bonferroni corrections was then adjusted accordingly. In addition, to validate the association findings discovered in the derivation cohort, we reperformed the association analysis in replication cohorts. The significance was defined using multiple-comparison tests with an FDR threshold of *P* < 0.05.

### Reporting summary

Further information on research design is available in the Nature Portfolio Reporting Summary linked to this article.

## Supplementary information

Supplementary Information STROBE-MR-checklist.

Reporting Summary

Supplementary Tables 1–39

## Source data

Source Data Fig. 2 Statistical source data.

Source Data Fig. 3 Statistical source data.

Source Data Fig. 4 Statistical source data.

Source Data Fig. 5 Statistical source data.

Source Data Fig. 6 Statistical source data.

Source Data Fig. 7 Statistical source data.

Source Data Fig. 8 Statistical source data.

Source Data Extended Data Fig. 2 Statistical source data.

Source Data Extended Data Fig. 3 Statistical source data.

Source Data Extended Data Fig. 4 Statistical source data.

Source Data Extended Data Fig. 5 Statistical source data.

Source Data Extended Data Fig. 6 Statistical source data.

Source Data Extended Data Fig. 7 Statistical source data.

Source Data Extended Data Fig. 8 Statistical source data.

Source Data Extended Data Fig. 9 Statistical source data.

Source Data Extended Data Fig. 10 Statistical source data.

## Data Availability

Detailed results of metabolite–disease and metabolite–trait associations, metabolite variations assessments, genetic associations, genetic colocalizations and disease discrimination have been deposited through an interactive portal and are publicly available (accessible at https://metabolome-phenome-atlas.com/). UK Biobank data are publicly available to bona fide researchers upon application at http://www.ukbiobank.ac.uk/using-the-resource/. This study was conducted using the UK Biobank under approved application numbers 202239 and 19542. FinnGen summary statistics are available through the FinnGen website (https://www.finngen.fi/en/access_results/). GWAS summary statistics for ref. ^44^ are publicly available through the NHGRI-EBI GWAS catalogue (GCST90301941–GCST90302173) and https://www.phpc.cam.ac.uk/ceu/lipids-and-metabolites/. UKB-derived metabolite and disease GWAS summary statistics are available to access through our web tool (https://metabolome-phenome-atlas.com/). Source data are provided with this paper.

## Code availability

All software used in this study is publicly available. Codes used for analysis can be accessible at https://github.com/jasonHKU0907/metabolome-phenome-atlas/.
