# Supplementary material for: Mapping the plasma metabolome to human health and disease in 274,241 adults
Source: Nat Metab. 2025 Sep 19;7(11):2366–84. doi: 10.1038/s42255-025-01371-1 (PMC12638258; doi:10.1038/s42255-025-01371-1)
Supplement: Supplementary file 1 — STROBE-MR-checklist. [file 42255_2025_1371_MOESM1_ESM.pdf]

nature metabolism

Supplementary information

https://doi.org/10.1038/s42255-025-01371-1

Mapping the plasma metabolome to human health and disease in 274,241 adults

In the format provided by the authors and unedited

STROBE-MR checklist of recommended items to address in reports of Mendelian randomization studies[1 2]

Item No.	Section		Checklist item	Page No.	Relevant text from manuscript
1	**TITLE and ABSTRACT**		Indicate Mendelian randomization (MR) as the study's design in the title and/or the abstract if that is a main purpose of the study	3	Mendelian randomization analyses provided support for causal relationships of 7,570 metabolite-disease pairs
	**INTRODUCTION**				
2	**Background**		Explain the scientific background and rationale for the reported study. What is the exposure? Is a potential causal relationship between exposure and outcome plausible? Justify why MR is a helpful method to address the study question	4	Metabolites, signifying a complex interplay between genotype, behaviour, and environment, provide a unique readout of human health and disease<br>metabolites are more closely tied to phenotypes due to their key roles in physiological function control.<br>Most metabolomic-based studies have typically been cross-sectional with case-control approaches biased by possible reverse causality.
3	**Objectives**		State specific objectives clearly, including pre-specified causal hypotheses (if any). State that MR is a method that, under specific assumptions, intends to estimate causal effects	5	Integrating genetic signals of metabolites and diseases, we inferred the potentially causal effects of metabolites upon diseases using Mendelian randomization (MR) analysis.
	**METHODS**				
4	**Study design and data sources**		Present key elements of the study design early in the article. Consider including a table listing sources of data for all phases of the study. For each data source contributing to the analysis, describe the following:		
		a)	Setting: Describe the study design and the underlying population, if possible. Describe the setting, locations, and relevant dates, including periods of recruitment, exposure, follow-up, and data collection, when available.	5, 34	The study analyzed 313 plasma metabolites linked to 1,386 diseases and 3,142 traits<br>Included 136,016-220,000 white British participants<br>Longitudinal follow-up for a median of 14.9 years<br>Used FinnGen study and UK Biobank data
		b)	Participants: Give the eligibility criteria, and the sources and methods of selection of participants. Report the sample size, and whether any power or sample size calculations were carried out prior to the main analysis	35	We accessed GWAS summary statistics for 368 diseases from FinnGen Release 10 (https://www.finngen.fi/en/access_results) and performed GWAS analysis for the remaining 107 diseases in a subset of 220,000 white British UKB participants (available at https://metabolome-phenome-atlas.com). Detailed source information

					is provided in Supplementary Tables 31–35 under "GWAS data source".<br><br>Metabolites' GWAS summary statistics were derived from a genome-wide meta-analysis of 233 NMR circulating metabolic traits, involving up to 136,016 participants from 33 cohorts. Among these, we included data of 223 metabolic traits that overlap with the metabolic profile analyzed in our study.
		c)	Describe measurement, quality control and selection of genetic variants	35	Instrumental variables were selected using PLINK 2.0 clumping function (clump-kb 500, clump-r2 0.1, clump-p1 5 × 10−8), and the European 1,000 Genomes phase 3 dataset was used as the reference genome.<br><br>F-statistics was calculated for all genetic instruments, implementing a threshold of F>10 to indicate strong instruments.
		d)	For each exposure, outcome, and other relevant variables, describe methods of assessment and diagnostic criteria for diseases	25-26, 28	The metabolic profiling of EDTA samples was performed through the Nightingale Health NMR biomarker platform using high-throughput NMR spectroscopy.<br><br>The diagnostic data in the UKB was obtained from UK Hospital Episode Statistics data. Disease endpoints were defined based on the first occurrence of the 3-character International Classification of Diseases (ICD)-10 code using the hospital inpatient records
		e)	Provide details of ethics committee approval and participant informed consent, if relevant	38	The study was conducted following the Declaration of Helsinki. The UK Biobank has research tissue bank approval from the North West Multi-Center Research Ethics Committee (11/NW/0382). Written informed consent was obtained from all participants. The present study was approved by UK Biobank under application numbers 202239 and 19542.
5	**Assumptions**		Explicitly state the three core IV assumptions for the main analysis (relevance, independence and exclusion restriction) as well assumptions for any additional or sensitivity analysis	35	For primary bidirectional MR analyses, instrumental variables were selected using PLINK 2.0 clumping function (clump-kb 500, clump-r2 0.1, clump-p1 5 × 10−8), and the European 1,000 Genomes phase 3 dataset was used as the reference genome. Additionally, we performed two sensitivity analyses for forward MR: (1) To minimize the potential influence of diet-related genetic variation, we

					excluded SNPs associated with dietary intake, which were identified from a GWAS on dietary habits in the UKB; (2) To minimize potential horizontal pleiotropy, we excluded genetic variants associated with more than five metabolites ($P<5\times10^{-8}$)
6	**Statistical methods: main analysis**		Describe statistical methods and statistics used		
		a)	Describe how quantitative variables were handled in the analyses (i.e., scale, units, model)	35	For each metabolite, values deviating by more than four times the interquartile range from the median were removed, followed by natural log transformation.
		b)	Describe how genetic variants were handled in the analyses and, if applicable, how their weights were selected	35	For primary bidirectional MR analyses, instrumental variables were selected using PLINK 2.0 clumping function (clump-kb 500, clump-r2 0.1, clump-p1 5 × 10−8), and the European 1,000 Genomes phase 3 dataset was used as the reference genome. Additionally, we performed two sensitivity analyses for forward MR: (1) To minimize the potential influence of diet-related genetic variation, we excluded SNPs associated with dietary intake, which were identified from a GWAS on dietary habits in the UKB; (2) To minimize potential horizontal pleiotropy, we excluded genetic variants associated with more than five metabolites ($P<5\times10^{-8}$)
		c)	Describe the MR estimator (e.g. two-stage least squares, Wald ratio) and related statistics. Detail the included covariates and, in case of two-sample MR, whether the same covariate set was used for adjustment in the two samples	35-36	For the main MR analyses, we employed two methods: the Wald ratio method for cases with a single genetic instrument, and the inverse variance weighted (IVW) method when multiple instruments were available"<br><br>For IVW analyses, we employed a multiplicative random-effects model due to observed heterogeneity across SNPs, allowing us to account for potential over-dispersion and variant-specific heterogeneity.<br><br>To ensure reliable causal inference, we implemented multiple robust MR methods as sensitivity analyses, specifically: MR-Egger, weighted median, simple mode, and weighted mode

					Different covariate adjustments were applied in the exposure and outcome GWAS. The disease GWAS from FinnGen adjusted for sex, age, genotyping batch and ten PCs. The metabolite GWAS used the same covariate set.
		d)	Explain how missing data were addressed	36	Missing data was handled through complete case analysis
		e)	If applicable, indicate how multiple testing was addressed	36	Results with q<0.05 after FDR multiple testing correction were considered significant.
7	**Assessment of assumptions**		Describe any methods or prior knowledge used to assess the assumptions or justify their validity	35-36	To handle genetic instruments associated with multiple metabolites, variants selected as instrumental variables for more than five metabolites were excluded to minimize potential horizontal pleiotropy<br>We conducted heterogeneity assessments through both IVW and Egger-based tests, and pleiotropy was assessed by MR-Egger intercepts<br>F-statistics was calculated for all genetic instruments, implementing a threshold of F>10 to indicate strong instruments
8	**Sensitivity analyses and additional analyses**		Describe any sensitivity analyses or additional analyses performed (e.g. comparison of effect estimates from different approaches, independent replication, bias analytic techniques, validation of instruments, simulations)	35-37	To ensure reliable causal inference, we implemented multiple robust MR methods as sensitivity analyses, specifically: MR-Egger, weighted median, simple mode, and weighted mode<br>We repeated the primary MR and colocalization analyses using this UKB-derived GWAS as a sensitivity analysis. For each metabolite, outliers outside four times the interquartile range were removed, followed by natural log transformation. A total of 189,846 white British participants with both metabolomics and genomic data were included. GWAS analysis was conducted using an additive linear regression model implemented in PLINK2.0. The covariates included age, ethnicity, sex, fasting time, month of assessment, genotype measurement batch, the top 40 genotype PCs, age indicators by sex interactions, and ethnicity by sex interactions. Metabolites' GWAS summary statistics are available to access through our webtool.

9	**Software and pre-registration**				
		a)	Name statistical software and package(s), including version and settings used	36	TwoSampleMR (v0.6.1) R package
		b)	State whether the study protocol and details were pre-registered (as well as when and where)	-	Not reported
	**RESULTS**				
10	**Descriptive data**				
		a)	Report the numbers of individuals at each stage of included studies and reasons for exclusion. Consider use of a flow diagram	35	Metabolites' GWAS summary statistics were derived from a genome-wide meta-analysis of 233 NMR circulating metabolic traits, involving up to 136,016 participants from 33 cohorts. Among these, we included data of 223 metabolic traits that overlap with the metabolic profile analyzed in our study.
		b)	Report summary statistics for phenotypic exposure(s), outcome(s), and other relevant variables (e.g. means, SDs, proportions)	16-17	Reported in "Mendelian randomization and colocalization prioritize causal metabolites" section and sTable31-35
		c)	If the data sources include meta-analyses of previous studies, provide the assessments of heterogeneity across these studies	-	Not applicable
		d)	For two-sample MR:<br>i. Provide justification of the similarity of the genetic variant-exposure associations between the exposure and outcome samples<br>ii. Provide information on the number of individuals who overlap between the exposure and outcome studies	23, 35	i. both of white British ethnicity<br>ii. no overlap
11	**Main results**				
		a)	Report the associations between genetic variant and exposure, and between genetic variant and outcome, preferably on an interpretable scale		We accessed FinnGen R10 GWAS statistics for 368 diseases (https://www.finngen.fi/en/access_results) and performed GWAS analysis for the remaining 107 diseases in a subset of 220,000 white British UKB participants.<br>Metabolites' GWAS summary statistics were derived from a genome-wide meta-analysis of 233 NMR circulating metabolic traits, involving up to 136,016 participants from 33 cohorts. Among these, we included data of 223 metabolic traits that

					overlap with the metabolic profile analyzed in our study.
		b)	Report MR estimates of the relationship between exposure and outcome, and the measures of uncertainty from the MR analysis, on an interpretable scale, such as odds ratio or relative risk per SD difference	27, 35	Moreover, phospholipids to total lipids in very large VLDL percentage (XL-VLDL-PL%) exhibited the largest effect size in increasing the risk of familial hypercholesterolemia (Odds ratio, OR=5.03 [3.97-6.38], q value=7.39×10-39), while free cholesterol to total lipids in small HDL percentage (S-HDL-FC%) was the most evident protective metabolite (OR=0.35 [0.30-0.40], q value=4.28×10-48) (Fig. 6d). Furthermore, total lipids in very small VLDL (XS-VLDL-L) exhibited the strongest association with increased risk of myocardial infarction (OR=1.55 [1.48-1.63], q value=1.56×10-64), while phospholipids to total lipids in small HDL percentage (S-HDL-PL%) (OR=0.63 [0.59-0.67], q value=7.03×10-48) exhibited the most notable protective effect (Fig. 6e).
		c)	If relevant, consider translating estimates of relative risk into absolute risk for a meaningful time period	-	Not applicable
		d)	Consider plots to visualize results (e.g. forest plot, scatterplot of associations between genetic variants and outcome versus between genetic variants and exposure)	-	Fig.6
12	**Assessment of assumptions**				
		a)	Report the assessment of the validity of the assumptions	36	We conducted heterogeneity assessments through both IVW and Egger-based tests, and pleiotropy was assessed by MR-Egger intercepts. F-statistics was calculated for all genetic instruments, implementing a threshold of F>10 to indicate strong instruments.
		b)	Report any additional statistics (e.g., assessments of heterogeneity across genetic variants, such as I^2, Q statistic or E-value)	36	We conducted heterogeneity assessments through both IVW and Egger-based tests, and pleiotropy was assessed by MR-Egger intercepts. F-statistics was calculated for all genetic instruments, implementing a threshold of F>10 to indicate strong instruments.
13	**Sensitivity analyses and additional analyses**			19	We conducted sensitivity analysis for MR and colocalization analysis using UKB-derived Metabolites’ GWAS dataset. Detailed results are shown in Supplementary Tables 37-39.

		a)	Report any sensitivity analyses to assess the robustness of the main results to violations of the assumptions	35-36	To ensure reliable causal inference, we implemented multiple robust MR methods as sensitivity analyses, specifically: MR-Egger, weighted median, simple mode, and weighted mode, with results presented in the Supplementary Table<br>We conducted heterogeneity assessments through both IVW and Egger-based tests, and pleiotropy was assessed by MR-Egger intercepts. F-statistics was calculated for all genetic instruments, implementing a threshold of F>10 to indicate strong instruments.
		b)	Report results from other sensitivity analyses or additional analyses	-	results presented in the sTable31-35
		c)	Report any assessment of direction of causal relationship (e.g., bidirectional MR)	17	Bidirectional MR was conducted
		d)	When relevant, report and compare with estimates from non-MR analyses	-	Not applicable
		e)	Consider additional plots to visualize results (e.g., leave-one-out analyses)	-	We have included Manhattan plots, forest plots, and bar plots to visualize the results.
	**DISCUSSION**				
14	**Key results**		Summarize key results with reference to study objectives		
15	**Limitations**		Discuss limitations of the study, taking into account the validity of the IV assumptions, other sources of potential bias, and imprecision. Discuss both direction and magnitude of any potential bias and any efforts to address them	23	The highly correlated nature of metabolites and pleiotropic effects of genetic variants also pose inherent challenges for MR analysis, despite our exclusion of highly pleiotropic variants and sensitivity analyses. Although multivariable MR has the potential to address these issues, simultaneously analyzing hundreds of metabolites remains computationally prohibitive with current methods. Further methodological developments are needed to better handle high-dimensional metabolomics data.
16	**Interpretation**				
		a)	Meaning: Give a cautious overall interpretation of results in the context of their limitations and in comparison with other studies	4, 5,16…	In interpreting the results, we used cautious language such as "potentially causal" and "provide support for causal relationships" to reflect the limitations and uncertainties inherent in the analysis.
		b)	Mechanism: Discuss underlying biological mechanisms that could drive a potential causal relationship between the investigated exposure and the outcome, and	21	Most potentially causal metabolite-disease pairs were found in metabolic diseases and CVD, with

			whether the gene-environment equivalence assumption is reasonable. Use causal language carefully, clarifying that IV estimates may provide causal effects only under certain assumptions		the well-known bidirectional causal relationship between albumin level and chronic kidney disease (CKD)38,39. Different medications targeting this metabolite have been and continue to be developed, demonstrating the remarkable promise of potentially causal metabolites in the treatment of diseases. Moreover, as indicated by the causal relationship between albumin and metabolic diseases revealed in this atlas, while lowering albumin levels may and metabolic syndrome revealed in this atlas, although lowering albumin can decrease CKD events, it might also increase the risk of metabolic diseases.
		c)	Clinical relevance: Discuss whether the results have clinical or public policy relevance, and to what extent they inform effect sizes of possible interventions	21	These findings suggest this atlas's value in identifying novel and safe targets for future pharmaceutical interventions towards a wide range of human diseases.
17	**Generalizability**		Discuss the generalizability of the study results (a) to other populations, (b) across other exposure periods/timings, and (c) across other levels of exposure	23	Finally, as our MR analyses were based on European populations, further studies are needed to establish the generalizability in diverse ancestral groups.
	**OTHER INFORMATION**				
18	**Funding**		Describe sources of funding and the role of funders in the present study and, if applicable, sources of funding for the databases and original study or studies on which the present study is based	36	This study was supported by grants from the STI2030-Major Projects (2022ZD0211600 to J.-T.Y.), National Natural Science Foundation of China (82071201, 82271471, and 92249305 to J.-T.Y.; 82071997 to W.C.), Shanghai Municipal Science and Technology Major Project (2023SHZDZX02 to J.-T.Y. and 2018SHZDZX01 to J.-F.F.), National Key Research and Development Program of China (2023YFC3605400 to W.C.), Shanghai Pujiang Talent Program (23PJD006 to J.Y.), Research Start-up Fund of Huashan Hospital (2022QD002 to J.-T.Y.), Excellence 2025 Talent Cultivation Program at Fudan University (3030277001 to J.-T.Y.), Shanghai Talent Development Funding for The Project (2019074 to J.-T.Y.), Shanghai Rising-Star Program (21QA1408700 to W.C.), 111 Project (B18015 to J.-F.F.), Humboldt Research Award (to J.-F.F.) and ZHANGJIANG LAB, Tianqiao and Chrissy Chen Institute, the State Key Laboratory of Neurobiology and Frontiers Center for Brain Science of Ministry

				of Education, and Shanghai Center for Brain Science and Brain-Inspired Technology, Fudan University.
19	**Data and data sharing**	Provide the data used to perform all analyses or report where and how the data can be accessed, and reference these sources in the article. Provide the statistical code needed to reproduce the results in the article, or report whether the code is publicly accessible and if so, where	38	All detailed results of metabolite-disease/-trait associations, life-/disease-span trajectories, genetic associations, genetic colocalizations, and prediction and diagnosis have been deposited through an interactive portal and are publicly available (accessible at https://metabolome-phenome-atlas.com/). UK Biobank data are publicly available to bona fide researchers upon application at http://www.ukbiobank.ac.uk/using-the-resource/. This study was conducted using the UK Biobank under approved application numbers 202239 and 19542.<br>All software used in this study is publicly available. Codes used for analysis can be accessible at https://github.com/jasonHKU0907/metabolome-phenome-atlas.
20	**Conflicts of Interest**	All authors should declare all potential conflicts of interest	39	The authors declared no potential conflicts of interest concerning the research, authorship, and/or publication of this article.

This checklist is copyrighted by the Equator Network under the Creative Commons Attribution 3.0 Unported (CC BY 3.0) license.

1. Skrivankova VW, Richmond RC, Woolf BAR, Yarmolinsky J, Davies NM, Swanson SA, et al. Strengthening the Reporting of Observational Studies in Epidemiology using Mendelian Randomization (STROBE-MR) Statement. JAMA. 2021;under review.

2. Skrivankova VW, Richmond RC, Woolf BAR, Davies NM, Swanson SA, VanderWeele TJ, et al. Strengthening the Reporting of Observational Studies in Epidemiology using Mendelian Randomisation (STROBE-MR): Explanation and Elaboration. BMJ. 2021;375:n2233.
