# Supplementary material for: Mapping the plasma metabolome to human health and disease in 274,241 adults
Source: Nat Metab. 2025 Sep 19;7(11):2366–84. doi: 10.1038/s42255-025-01371-1 (PMC12638258; doi:10.1038/s42255-025-01371-1)
Supplement: Supplementary file 2 — Reporting Summary [file 42255_2025_1371_MOESM2_ESM.pdf]

nature portfolio

Corresponding author(s): Jin-Tai Yu, Wei Cheng, Jian-Feng Feng

Last updated by author(s): Feb 18, 2025

Reporting Summary

Nature Portfolio wishes to improve the reproducibility of the work that we publish. This form provides structure for consistency and transparency in reporting. For further information on Nature Portfolio policies, see our Editorial Policies and the Editorial Policy Checklist.

Statistics

For all statistical analyses, confirm that the following items are present in the figure legend, table legend, main text, or Methods section.

n/a	Confirmed	
☐	☒	The exact sample size (*n*) for each experimental group/condition, given as a discrete number and unit of measurement
☐	☒	A statement on whether measurements were taken from distinct samples or whether the same sample was measured repeatedly
☐	☒	The statistical test(s) used AND whether they are one- or two-sided<br>*Only common tests should be described solely by name; describe more complex techniques in the Methods section.*
☐	☒	A description of all covariates tested
☐	☒	A description of any assumptions or corrections, such as tests of normality and adjustment for multiple comparisons
☐	☒	A full description of the statistical parameters including central tendency (e.g. means) or other basic estimates (e.g. regression coefficient) AND variation (e.g. standard deviation) or associated estimates of uncertainty (e.g. confidence intervals)
☐	☒	For null hypothesis testing, the test statistic (e.g. *F*, *t*, *r*) with confidence intervals, effect sizes, degrees of freedom and *P* value noted<br>*Give P values as exact values whenever suitable.*
☒	☐	For Bayesian analysis, information on the choice of priors and Markov chain Monte Carlo settings
☐	☒	For hierarchical and complex designs, identification of the appropriate level for tests and full reporting of outcomes
☐	☒	Estimates of effect sizes (e.g. Cohen's *d*, Pearson's *r*), indicating how they were calculated

Our web collection on statistics for biologists contains articles on many of the points above.

Software and code

Policy information about availability of computer code

Data collection	No software was involved in data collection (data used is all directly available from UK Biobank, as described in detail in the paper
Data analysis	The following software and packages were used for data analysis: Python (https://www.python.org/), GCTA v1.94.0 (https://yanglab.westlake.edu.cn/software/gcta/) , and R v.4.2.0 (https://www.r-project.org/). All software used in this study is publicly available. Codes used for analysis can be accessed at https://github.com/jasonHKU0907/metabolome-phenome-atlas.

For manuscripts utilizing custom algorithms or software that are central to the research but not yet described in published literature, software must be made available to editors and reviewers. We strongly encourage code deposition in a community repository (e.g. GitHub). See the Nature Portfolio guidelines for submitting code & software for further information.

Data

Policy information about availability of data

All manuscripts must include a data availability statement. This statement should provide the following information, where applicable:

- Accession codes, unique identifiers, or web links for publicly available datasets
- A description of any restrictions on data availability
- For clinical datasets or third party data, please ensure that the statement adheres to our policy

All detailed results of metabolite-disease/-trait associations, metabolic variations assessments, genetic associations, genetic colocalizations, and disease discrimination have been deposited through an interactive portal and are publicly available (accessible at https://metabolome-phenome-atlas.com/). UK Biobank

data are publicly available to bona fide researchers upon application at http://www.ukbiobank.ac.uk/using-the-resource/. This study was conducted using the UK Biobank under approved application numbers 202239 and 19542.

Research involving human participants, their data, or biological material

Policy information about studies with human participants or human data. See also policy information about sex, gender (identity/presentation), and sexual orientation and race, ethnicity and racism.

Reporting on sex and gender	We used UK Biobank field 31 (Sex), which contains both NHS-recorded and self-reported sex information. In line with the SAGER guidelines, we have clarified the definition of sex differences in the manuscript, ensuring that we distinguish biological sex from gender and carefully report findings in the context.
Reporting on race, ethnicity, or other socially relevant groupings	The ethnic information was extracted from the UK Biobank Field 21000, which was self-reported during participants' initial visit as part of the touchscreen questionnaire. We used "self-reported ethnic background" throughout the manuscript to ensure clarity and accuracy.
Population characteristics	This study included 274,241 participants from the UKB with Nuclear Magnetic Resonance (NMR) metabolic measures. Analyzed participants had a median age of 58.0 (interquartile range, IQR, 50.0-63.0) years at the time of blood sample collection, of whom 54.0% (n=147,994) were females, and 95.1% (n=260,800) were White ancestry. Until November 2023, participants had a median follow-up of 14.9 (IQR, 14.1-15.5) years.
Recruitment	This study adopted participants recruited from the UKB, a community-based cohort comprising adults aged between 40 and 69 years. Over 500,000 participants were recruited from 22 assessment centers across the UK between 2006 and 2010, and they were all registered with the UK National Health Service. This study included 274,241 participants who underwent metabolic profiling of their blood plasma samples collected during the baseline visits. The assessment visits comprised interviews and questionnaires covering lifestyles and health conditions, physical measures, biological samples, imaging, and genotype. The database is linked to national heath datasets, including primary care,hospital inpatient, death,and cancer registration data.
Ethics oversight	The study was conducted following the Declaration of Helsinki, and ethnic approval was obtained from the North West Multi-centre Research Ethics Committee (MREC, https://www.ukbiobank.ac.uk/learn-more-about-uk-biobank/about-us/ethics). All study participants provided informed consent. This research was carried out using the UKB Resource under approved application numbers 202239 and 19542.

Note that full information on the approval of the study protocol must also be provided in the manuscript.

Field-specific reporting

Please select the one below that is the best fit for your research. If you are not sure, read the appropriate sections before making your selection.

☒ Life sciences ☐ Behavioural & social sciences ☐ Ecological, evolutionary & environmental sciences

For a reference copy of the document with all sections, see nature.com/documents/nr-reporting-summary-flat.pdf

Life sciences study design

All studies must disclose on these points even when the disclosure is negative.

Sample size	No statistical methods were used to predetermine sample sizes. This study included 274,241 participants who underwent metabolic profiling of their blood plasma samples collected during the baseline visits.
Data exclusions	Participants without metabolite measurement data were excluded.
Replication	We split the data into 148,974 White ancestry individuals released in Phase 2 (assessed between April 2020 and June 2022) as a derivation cohort, 111,826 White ancestry individuals released in Phase 1 (assessed between June 2019 and April 2020) as replication cohort 1 and 13,444 non-White ancestry individuals from both phases as replication cohort 2. We conducted replication analysis of metabolite-phenotype associations separately in White and non-White ancestry groups, using a significant threshold determined by Bonferroni correction, consistent with the approach used in the discovery cohort.
Randomization	There is nothing in this study that pertains to randomization. We are using existing data released by UK Biobank. UK Biobank is an observational prospective epidemiological study, and our study use all available subjects that fulfill the criteria described above. Hence there is no equivalent process of randomization that comes into this analysis (this is not a controlled randomized study).
Blinding	Blinding was not applicable to this study.

Reporting for specific materials, systems and methods

We require information from authors about some types of materials, experimental systems and methods used in many studies. Here, indicate whether each material, system or method listed is relevant to your study. If you are not sure if a list item applies to your research, read the appropriate section before selecting a response.

Materials & experimental systems

n/a	Involved in the study
☒	☐ Antibodies
☒	☐ Eukaryotic cell lines
☒	☐ Palaeontology and archaeology
☒	☐ Animals and other organisms
☒	☐ Clinical data
☒	☐ Dual use research of concern
☒	☐ Plants

Methods

n/a	Involved in the study
☒	☐ ChIP-seq
☒	☐ Flow cytometry
☐	☒ MRI-based neuroimaging

Plants

Seed stocks

Report on the source of all seed stocks or other plant material used. If applicable, state the seed stock centre and catalogue number. If plant specimens were collected from the field, describe the collection location, date and sampling procedures.

Novel plant genotypes

Describe the methods by which all novel plant genotypes were produced. This includes those generated by transgenic approaches, gene editing, chemical/radiation-based mutagenesis and hybridization. For transgenic lines, describe the transformation method, the number of independent lines analyzed and the generation upon which experiments were performed. For gene-edited lines, describe the editor used, the endogenous sequence targeted for editing, the targeting guide RNA sequence (if applicable) and how the editor was applied.

Authentication

Describe any authentication procedures for each seed stock used or novel genotype generated. Describe any experiments used to assess the effect of a mutation and, where applicable, how potential secondary effects (e.g. second site T-DNA insertions, mosiacism, off-target gene editing) were examined.

Magnetic resonance imaging

Experimental design

Design type

Our analysis comprised 2,151 imaging traits, spanning various modalities such as brain imaging (including T1 structural brain MRI, susceptibility-weighted brain MRI, and diffusion brain MRI) (n=1978), as well as cardiovascular magnetic resonance (CMR) (n=129) and abdominal MRI traits (n=44). Image acquisition followed predefined standard operating procedures using uniform equipment and staff training. Specifically, multi-modal brain MRIs were conducted using Siemens 3T scanners following an extensive data processing and quality control pipeline, CMR and abdominal scans were performed using Siemens 1.5T scanners with detailed protocols and processing described earlier.

Design specifications

Not applicable, as our analyses did not use any functional MRI data.

Behavioral performance measures

Behavioral performance in the MRI scanner was not used in this study.

Acquisition

Imaging type(s)

T1 structural brain MRI, susceptibility-weighted brain MRI, and diffusion brain MRI (n=1978), as well as cardiovascular magnetic resonance (CMR) (n=129) and abdominal MRI traits (n=44).

Field strength

Specifically, multi-modal brain MRIs were conducted using Siemens 3T scanners following an extensive data processing and quality control pipeline, CMR and abdominal scans were performed using Siemens 1.5T scanners with detailed protocols and processing described earlier.

Sequence & imaging parameters

(1) T1-weighted structural imaging
Resolution: 1x1x1 mm
Field-of-view: 208x256x256 matrix
Duration: 5 minutes
3D MPRAGE, sagittal, in-plane acceleration iPAT=2, prescan-normalise
(2) Diffusion imaging
Resolution: 2x2x2 mm
Field-of-view: 104x104x72 matrix
Duration: 7 minutes (including 36 seconds phase-encoding reversed data)
5x b=0 (+3x b=0 blip-reversed), 50x b=1000 s/mm2, 50x b=2000 s/mm2
Gradient timings: δ=21.4 ms, Δ=45.5 ms; Spoiler b-value = 3.3 s/mm2
SE-EPI with x3 multislice acceleration, no iPAT, fat saturation
(3) Susceptibility-weighted structural imaging
Resolution: 0.8x0.8x3 mm
Field-of-view: 256x288x48 matrix
Duration: 2.5 minutes
Two echos, TE=9.42,20 ms
3D, axial, in-plane acceleration iPAT=2, partial Fourier = 7/8, prescan-normalise
(4) Cardiovascular Magnetic Resonance (CMR)

Resolution: 1.6 x 1.6 x 6.0 mm (short axis cines).
Field-of-View: 380 x 380 mm.Duration: ~20 minutes (part of a 30-minute combined protocol).
Pulse Sequence: TRUFI (Turbo Gradient Echo) for most sequences.
Acquisition Views: Sagittal, coronal, and transverse; long-axis cines (HLA, VLA, LVOT).
ECG Gating: Retrospective gating.
Additional Sequences: ShMOLLI for myocardial T1 mapping, phase contrast for aortic flow.
(5) Abdominal MRI
Resolution: 8mm slice for MOLLI, 6mm for multi-gradient echo.
Field-of-View: 400 x 400 mm.
Duration: ~3 minutes for LiverMultiScan.
Pulse Sequence: MOLLI for T1-mapping, multi-gradient echo for fat/water fraction.
Acquisition Views: Transverse slice at the porta hepatis for liver imaging.
Data Processing: Liver fat, iron, and inflammation measures processed by Perspectum Diagnostics.

Area of acquisition

For Brain Imaging, the acquisition covers the whole-brain. For Cardiovascular Magnetic Resonance (CMR), the area of acquisition covers the chest and abdomen with partial coverage using sagittal, coronal, and transverse views, assessing cardiac function and aortic compliance. For Abdominal MRI, the focus is primarily on the liver, with a transverse slice at the porta hepatis for T1 imaging, along with additional imaging of the pancreas and other abdominal structures.

Diffusion MRI ☒ Used ☐ Not used

Parameters

Number of directions: 30 directions, b-values: b=1000 s/mm², b=2000 s/mm². Single-shell or multi-shell: Single-shell. Cardiac gating: Not used

Preprocessing

Preprocessing software

Multi-modal brain MRIs were conducted using Siemens 3T scanners following an extensive data processing and quality control pipeline[1,2], CMR and abdominal scans were performed using Siemens 1.5T scanners with detailed protocols and processing described earlier[3-5].
1. Miller, K. L. et al. Multimodal population brain imaging in the UK Biobank prospective epidemiological study. Nat. Neurosci. 19, 1523–1536 (2016).
2. F, A.-A. et al. Image processing and Quality Control for the first 10,000 brain imaging datasets from UK Biobank. NeuroImage 166, (2018).
3. Bai, W. et al. A population-based phenome-wide association study of cardiac and aortic structure and function. Nat. Med. 26, 1654–1662 (2020).
4. Mojtahed, A. et al. Reference range of liver corrected T1 values in a population at low risk for fatty liver disease-a UK Biobank sub-study, with an appendix of interesting cases. Abdominal Radiology (New York) 44, 72–84 (2019).
5. Langner, T. et al. Kidney segmentation in neck-to-knee body MRI of 40,000 UK Biobank participants. Sci. Rep. 10, 20963 (2020).

Normalization

Quantitative susceptilbility mapping (QSM) spatial maps were transformed using FNIRT (part of FSL) to a 1-mm T1 template in MNI-space, transformations were provided by the UK Biobank brain processing pipeline (as described in Alfaro-Almagro et al. 2018).

Normalization template

MNI152 1mm standard space

Noise and artifact removal

Noise and artifact removal was performed based on Wang et al., 2022

Volume censoring

No volume censoring was performed to imaging data.

Statistical modeling & inference

Model type and settings

The associations between metabolites and imaging traits were assessed using various regression models tailored to the specific types of traits. Linear regressions were adopted to test continuous and binary traits, while proportional odds logistic regression was applied for ordered categorical traits. All models adjusted the same set of covariates as aforementioned. Bonferroni correction was applied for multiple tests comparison (P<0.05/[313*3,142]).

Effect(s) tested

Not applicable

Specify type of analysis: ☒ Whole brain ☐ ROI-based ☐ Both

Statistic type for inference

(See Eklund et al. 2016)

Not applicable

Correction

Bonferroni correction was applied for multiple tests comparison (P<0.05/[313*3,142]).

Models & analysis

n/a	Involved in the study
☒	☐ Functional and/or effective connectivity
☒	☐ Graph analysis
☐	☒ Multivariate modeling or predictive analysis

Multivariate modeling and predictive analysis	The associations between metabolites and imaging traits were assessed using various regression models tailored to the specific types of traits. Linear regressions were adopted to test continuous and binary traits, while proportional odds logistic regression was applied for ordered categorical traits. All models adjusted the same set of covariates as aforementioned.
